# Deterministic segmentation of grains in dense Laue microdiffraction datasets

**DOI:** 10.1107/S1600576726007995

**Published:** 2026-09-25

**Authors:** Sergio Bongiorno, Jean-Sébastien Micha, Samuel Tardif, Joël Eymery

**Affiliations:** a Univ. Grenoble Alpes, CEA, IRIG, MEM, NRX, 38000 Grenoble, France; b Univ. Grenoble Alpes, CNRS, UMR SyMMES, CEA, IRIG, 38000 Grenoble, France; DESY, Hamburg, Germany

**Keywords:** Laue microdiffraction, polycrystalline materials, grain segmentation, appearance maps, SiC

## Abstract

Although Laue microdiffraction is an essential non-destructive technique for probing crystallographic orientation and strain at submicrometre resolution, the extreme density of overlapping Bragg reflections often hinders its application to polycrystalline materials. We present a novel analysis pipeline, implemented in the open-source Python package *graintools*, designed to segment these complex datasets without prior knowledge of the microstructure. The workflow uses a multi-round peak search to handle diverse spot morphologies and constructs a look-up table to identify recurring reflections across raster-scan positions. These reflections are organized into spot families and represented as 2D binary appearance maps, which indicate the spatial occurrence of specific grains. To enhance data fidelity, connected component analysis is applied to clean these maps before they are grouped into clusters using cosine similarity. The method was successfully validated on a 3C-SiC polycrystalline sample, demonstrating its ability to isolate grain footprints, extract accurate orientations and reconstruct intra-grain deviatoric strain maps. This deterministic approach effectively circumvents the combinatorial and memory bottlenecks of traditional indexing and dictionary-based methods, providing a robust solution for analyzing highly populated diffraction patterns.

## Abstract 2

This work introduces a deterministic methodology for segmenting individual grain contributions from complex high-density Laue microdiffraction datasets by leveraging spatial correlations and clustering of peak appearance maps.

## 1. Introduction

The properties of polycrystalline materials are strongly determined by their microstructure. Grain size, boundaries, grain orientation and associated strain fields play a decisive role in heat flow, electron scattering, and thin films’ adhesion or delamination. In technologically relevant systems, particularly when grain sizes are of the order of micrometres, the microstructure becomes a key factor in determining device performance and mechanics. Accurate characterization in such cases requires spatially resolved probes capable of identifying local orientation and strain across many grains (Shukla *et al.*, 2024 ▸; Xu *et al.*, 2025 ▸).

Electron-based orientation mapping, most notably electron backscatter diffraction (EBSD) in a scanning electron microscope, is the natural first option because it routinely delivers high-quality phase and orientation maps with relatively high throughput. The limitation is that EBSD is intrinsically surface sensitive, as the signal originates from a near-surface volume and, therefore, the diffraction pattern reflects only the top few tens of nanometres of material under standard conditions. As a result, EBSD cannot directly access the crystallographic state inside the bulk of a polycrystal without resorting to destructive 3D strategies, such as serial sectioning (Echlin *et al.*, 2020 ▸; DeMott *et al.*, 2021 ▸). Such approaches trade volume coverage for long acquisition times, demanding preparation and potential alignment artifacts – constraints that are difficult to reconcile with non-destructive or *in situ* studies. High-angular-resolution EBSD can be used for quantitative strain mapping. It measures small lattice rotations and deviatoric elastic strains with high sensitivity by cross-correlating diffraction patterns (Britton *et al.*, 2013 ▸). However, it remains a near-surface measurement. These constraints motivate the use of X-ray methods when the objective is to obtain spatially resolved orientation and strain information across many grains within the bulk of a polycrystalline sample.

Several X-ray imaging and diffraction methods have been developed for polycrystalline materials and, in principle, could be applied to this problem. Although scanning monochromatic microdiffraction can probe the reciprocal space locally (Henningsson *et al.*, 2020 ▸; Etzelstorfer *et al.*, 2014 ▸; Zatterin *et al.*, 2025 ▸), the technique is impractical when thousands of grains are present in the illuminated volume because each grain must be sequentially brought into the Bragg condition. Coherent diffraction imaging and its Bragg variant can provide reconstructions of lattice distortions with nanometre resolution (Grimes *et al.*, 2024 ▸; Atlan *et al.*, 2023 ▸). Still, they require that the grains be isolated and aligned at a single Bragg condition to avoid overlap and achieve convergence of the phase reconstruction. Diffraction contrast tomography yields three-dimensional grain maps and, in some cases, orientation information. However, this method is optimized for larger grain sizes. While it captures shapes and average orientations, it generally does not resolve strain at the micrometre scale (Reischig *et al.*, 2013 ▸; Vaughan *et al.*, 2020 ▸). Dark-field X-ray microscopy offers submicrometre sensitivity to strain fields over extended regions, yet only around a selected Bragg reflection, making it unsuitable for comprehensive mapping of multiphase or highly polycrystalline samples (Yildirim *et al.*, 2025 ▸).

In this context, Laue microdiffraction has established itself as a versatile X-ray technique for probing crystallographic orientation and strain in crystalline materials with submicrometre spatial resolution (de Goes Foschiani *et al.*, 2026 ▸). Using an X-ray polychromatic beam, a large number of Bragg reflections are excited simultaneously, producing diffraction patterns of polycrystalline materials containing up to thousands of Laue spots. This makes it uniquely suited for the study of complex polycrystalline materials, which conventional monochromatic or Bragg-aligned techniques cannot effectively analyze. These techniques either require aligning each grain into a diffraction condition or are limited to a single reflection per grain. In contrast, Laue microdiffraction captures the crystallographic state of multiple grains in a single exposure, enabling the construction of orientation and strain maps without prior knowledge of the microstructure. Its non-destructive character and compatibility with *in situ* experiments have led to its adoption across a broad range of materials science problems (de Goes Foschiani *et al.*, 2026 ▸; Magalhaes *et al.*, 2025 ▸; Fréville *et al.*, 2026 ▸; Sanchez *et al.*, 2014 ▸).

The analytical challenge lies in the sheer density of peaks: a typical diffraction pattern of a polycrystalline material may contain several thousand spots, many of which overlap or display complex morphologies due to strain gradients, defects or the superposition of patterns from multiple grains. Accurate characterization of peak position, shape and intensity is a prerequisite for reliable indexing and strain refinement. Several computational approaches have been developed to address this problem. Classical methods rely on the geometric relationships between experimental spot positions and theoretical predictions, using either angle-based triplet matching (Tamura *et al.*, 2003 ▸; Tamura, 2014 ▸) or look-up tables of inter-spot distances (Ohba *et al.*, 1981 ▸; Micha *et al.*, 2025 ▸). These methods are computationally efficient for single crystals or sparse patterns but scale poorly when confronted with superimposed patterns containing tens of grains. Dictionary-based approaches, including dictionary-branch-bound methods (Seret *et al.*, 2022 ▸), extend applicability to more complex cases. These methods match experimental patterns against large simulated databases of orientations. Although this improves robustness against missing or spurious spots, it requires significant computational time and memory usage.

Machine learning has more recently emerged as a promising alternative. Convolutional neural networks have been trained to recognize and refine Laue spot morphologies, achieving sub-pixel accuracy in peak localization and accelerating the analysis pipeline (Kirstein *et al.*, 2023 ▸). Other approaches, such as LaueNN (Purushottam Raj Purohit *et al.*, 2022 ▸), use feed-forward neural networks to directly predict the Miller indices of reflections, enabling the rapid indexing of patterns containing hundreds of overlapping spots. Data-driven clustering and unsupervised learning have also been proposed to process entire raster scans of Laue patterns without full indexing, segmenting regions of interest in fatigued or defect-rich samples (Song *et al.*, 2019 ▸; Rong *et al.*, 2022 ▸). These methods significantly reduce the bottleneck of the indexing step, but they are typically validated using datasets containing up to approximately 2000 peaks per pattern. This validation process uses extensive synthetic training sets tailored to specific symmetries or experimental geometries.

Diffraction patterns containing thousands of peaks exceed the intended range and use of existing algorithms. Conventional indexing methods become computationally prohibitive due to the combinatorial explosion of possible matches, while dictionary-based approaches encounter excessive memory and runtime requirements. Neural-network-based models, although extremely fast once trained, require carefully curated training datasets, whose generation requires an estimate of the number of grains as well as their phase. As a result, a different strategy is needed to extract reliable structural information from these highly populated Laue patterns. This work proposes a new methodology for separating the Laue pattern into contributions from the different illuminated grains. This methodology is based on first principles and leverages the spatial correlation between Laue patterns recorded at adjacent scan points.

Silicon carbide (SiC) is a particularly relevant system for demonstrating advanced Laue microdiffraction analysis because it is a technologically critical wide-bandgap semiconductor for high-voltage, high-temperature and high-power-density applications, where device efficiency and reliability are tightly coupled to the crystal quality of the substrate and epilayers (Willander *et al.*, 2006 ▸; She *et al.*, 2017 ▸). In practical wafers and engineered stacks, the crystallographic state is rarely ideal: residual strain fields inherited from growth and processing, dislocation networks, and local defect populations can vary at the micrometre scale, leading to spatially heterogeneous electrical behavior and providing pathways for degradation mechanisms such as stacking-fault expansion driven by basal-plane dislocations (Tsuchida *et al.*, 2008 ▸; Konishi *et al.*, 2013 ▸; Kato *et al.*, 2022 ▸). Interfaces – most notably the SiO_2_/SiC system in MOS technologies but more generally any bonded or deposited layer – introduce further structural complexity through mismatch strain, near-interface defect states and local stress concentrations that can dominate functional performance (Fiorenza *et al.*, 2019 ▸; Wang & Jiang, 2020 ▸). These issues are exacerbated in polycrystalline or hybrid architectures where grain orientation, grain boundaries and strain gradients add additional sources of variability (Li *et al.*, 2025 ▸; Biard *et al.*, 2023 ▸; Huang *et al.*, 2025 ▸), motivating spatially resolved crystallographic probes capable of mapping orientation and strain across many grains and across interfaces in a non-destructive manner.

## 2. Methodology

Measurements were performed on the recently upgraded BM32 Laue microdiffraction setup (Ulrich *et al.*, 2011 ▸) at the European Synchrotron Radiation Facility (ESRF), using 3C-SiC polycrystalline wafer samples that were approximately 700 µm thick. The beamline X-ray source is a bending magnet (0.85 T) with a flat energy distribution from 5 to 27 keV, and the beam is micro-focused by Kirkpatrick–Baez mirrors to get a 300 × 400 nm^2^ (H × V) footprint. A 2D sCMOS detector (2018 × 2016 pixels), located 80 mm above the sample, collects the resulting diffraction patterns. The scanned area is here 50 × 50 µm^2^ in steps of 0.5 µm in both directions. It yields a dataset of 10201 images acquired with a counting time of 1 s. An example of the kind of images that are collected is shown in Fig. 1 ▸.

As schematized in Fig. 2 ▸, the current analysis method is based on the observation that each grain’s Laue pattern is limited to specific scan positions. Recovering the locations of all the detected diffraction peaks across the scan enables grouping of peaks that appear in the same region of the sample. This yields the single-crystal Laue pattern of each grain, along with its in-plane position and lateral extent, without prior indexing. Once these patterns are isolated, indexing becomes much simpler, as no extra peaks coming from other grains can hinder peak identification. Finally, orientation and elastic strain can be extracted using the usual methods.

Although conceptually simple, implementing this method requires handling the large volume of data and high spot density of individual images, all while keeping the code readable and easy for the community to adopt and maintain. For these reasons, it is implemented in Python. When appropriate, we use parallel computing approaches and just-in-time compilers to reduce computation time.

The following sections outline the ideas that informed the segmentation process. The supplementary materials provide a detailed description of its implementation.

### 2.1. Peak search

Peak detection in the raw detector images is a standard first step in Laue pattern processing. Following the Gaussian fit of the 2D intensity profile of detected local maxima, it provides sub-pixel peak positions alongside shape descriptors, such as peak intensity, full-widths at half maximum along the principal axes, inclination and deviation of the fitted maximum from the initial guess (given by the local maxima of intensity).

The initial objective is to maximize the number of correctly identified peaks while ensuring the quality of the resulting peak list – that is, the position, intensity and shape descriptors fitted for each peak – so that the downstream segmentation operates on trustworthy inputs.

Because the images contain peaks of varying size and morphology, and because neighboring peaks can lie in proximity, a single parameter set is often insufficient. Multiple rounds of peak detection are performed using different box sizes to determine the size of the window used to fit the intensity. A narrow window allows for the detection of small peaks when larger peaks are present in the immediate vicinity, especially if they are more intense. This procedure inevitably generates duplicate fits for the same physical spot location, and peaks that fall within a small distance threshold, typically no more than a few pixels, are removed. Additionally, peaks can be filtered using their descriptors for quality control purposes. For instance, an excessive deviation between the fitted and initial estimated peak positions can indicate a misfit, distorted peak or partial overlap.

The output of this stage (*i.e.* the input of the subsequent stage) is a set of peak lists containing the fitting results of the peaks in each image.

### 2.2. Peak tracking

#### 2.2.1. Clustering from a detector look-up table

In principle, one could compare the position of a peak across the dataset with those found in every image to identify where it appears. In practice, a direct pairwise search is prohibitive. With approximately 2000 peaks per image and 101 × 101 images, the naive approach would require processing about 20 million peak observations. This would increase the number of comparisons and generate substantial redundancy.

The data’s intrinsic properties suggest a reduction in complexity. Within any region where a given grain is illuminated, consecutive Laue patterns are nearly identical. The same reflections recur at nearly the same detector coordinates and are separated by only modest shifts induced by local misorientation or elastic strain. For tracking purposes, if a grain contributes *N*_R_ reflections and spans *N*_G_ scan positions, the dataset contains *N*_R_*N*_G_ observations that are simply manifestations of *N*_R_ nominal positions. Treating all *N*_R_*N*_G_ observations as independent multiplies the comparison count by *N*_G_, slows the computation and yields information that must later be consolidated.

To address this challenge, a look-up table (LUT) of nominal detector coordinates is constructed by pooling all peak positions from the raster and replacing those that lie within a small positional tolerance, deemed to represent the same reflection, by their average coordinate. Section S4 of the supplementary materials contains details about the algorithm and explains how to use it. Conceptually, the LUT enumerates the representative positions of recurring reflections across the raster, regardless of how frequently they are observed. This is illustrated in Fig. 3 ▸. Plotting all detected peaks reveals dense clouds around well-defined coordinates; the LUT replaces each cloud with a smaller set of nominal positions.

#### 2.2.2. Tracking against the LUT

The implementation of a LUT has been shown to significantly improve tracking by converting it into a search around physically meaningful coordinates. For each LUT entry, the task is to locate any detected peak across the raster that lies within a specified tolerance of that position. When a match is found, the image index and the fitted descriptors inherited from the detection stage (*e.g.* intensity, FWHM, inclination) are recorded. Collecting these rows for a given LUT position yields a spot family – a compact history of that reflection over the scan. If multiple peaks fall within tolerance in the same image, the ambiguity is resolved deterministically, by selecting either the nearest in position or, if specified, the one with the intensity closest to a prior match.

At the end of this stage, the dataset is reorganized into a list of spot families, one per LUT entry. This representation supplants millions of individual spots with a physically grounded catalog of reflections and their occurrences, thereby establishing the foundation for subsequent steps.

### 2.3. Appearance maps

Given the image indices stored in each spot family, the occurrence of a reflection over the scan can be represented as a two-dimensional binary image indicating its presence or absence at each raster position. This appearance map pinpoints the location of a peak in the sample, with the associated descriptors still accessible in the corresponding spot family. More precisely, since the beam illuminates a column of grains along its path [Fig. 1 ▸(*a*)], the appearance map represents the projection of the diffracting grain along the beam direction, *i.e.* its 2D footprint. The idea guiding the remainder of the segmentation is straightforward: spot families sharing the same or highly similar appearance maps belong to reflections originating from the same grain. The subsequent task is therefore to group appearance maps by similarity.

Fig. 4 ▸(*a*) shows a typical appearance map for an arbitrary peak. As is visible, this peak appears in a portion of the scanned area that can be easily recognized as a grain, and outside of it, there are small isolated regions that light up. These regions arise when reflections from other grains fall within the tolerance of the LUT entry, and with thousands of peaks per image – or, equivalently, a substantial illuminated volume – they are expected. However, because they occur randomly, it is harder to group similar maps together. To solve this issue, each appearance map goes through connected component analysis (CCA) under the eight-neighbor con­nectivity rule before grouping (Shapiro, 1996 ▸). The procedure labels all connected regions [Fig. 4 ▸(*b*)]. Retaining only the largest connected component yields an appearance map that isolates the grain of interest [Fig. 4 ▸(*c*)]. These maps form the basis for the final clustering step.

### 2.4. Appearance map matching

The process of clustering involves measuring the similarity between pairs of appearance maps and then grouping those that exceed a certain threshold. The similarity metric must capture the degree of spatial overlap while also penalizing mismatches in size. For instance, two grains with an apparent ‘perfect overlap’ arising when a small grain lies entirely within a much larger one should not be treated as equivalent. To satisfy these requirements with minimal computational cost, cosine similarity is used. Each pair of appearance maps is flattened into binary vectors **u** and **v**, and the similarity lying in the [0, 1] range is computed as 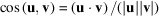.

Partial overlaps yield intermediate scores, identical maps return 1, and the case of perfect overlap with different sizes is down-weighted by the factor 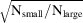, where *N*_small_ and *N*_large_ are the areas of the small grain and the larger one that encloses it, respectively.

Clustering is performed by thresholding the similarity. The cosine similarity is calculated for each appearance map in relation to all the others. Those that exceed the user-defined threshold are grouped. The implementation of this procedure is designed to assign the appearance maps to only one cluster. These relations can be interpreted as a similarity graph. In this graph, the appearance maps are the vertices, and the edges connect elements within the same cluster. Fig. 5 ▸ reports some elements within a given cluster.

### 2.5. Segmentation result

Although the segmentation formally ends with the clustering step, the output is reorganized so that each grain is represented by (i) a single grain appearance map covering its footprint and (ii) a grain average diffraction pattern sum­marizing its reflections. The latter is obtained by aggregating all peak families from the appearance maps assigned to the grain and for each one computing the average peak position and shape descriptors. These outputs serve as inputs for subsequent analyses, where needed (see Sections 2.6 and 3.3).

Fig. 6 ▸ reports representative outputs of the segmentation. Panels (*a*)–(*e*) show the grain appearance maps (*i.e.* the grain footprints); panels (*f*)–(*j*) display the corresponding grain average diffraction patterns. For each case, an orientation matrix was extracted from the grain average pattern using *LaueTools* (Micha *et al.*, 2025 ▸), and the theoretical Laue reflections computed from that orientation are overlaid in orange in Figs. 6 ▸(*f*)–(*j*). The overlays show that only a small number of peaks are spuriously attributed to a grain, and these are readily recognized and rejected during indexing of the averaged pattern. Importantly, even sparsely populated patterns, such as Fig. 6 ▸(*j*) with only seven reflections, yield the correct orientation.

Taken together, these examples show that the segmentation delivers both grain-related descriptors of the sample (footprints, number, orientations) and inputs suitable for subsequent analyses, such as grain statistics and strain refinement.

### 2.6. Validation

To validate the results, the grain orientation determined in Fig. 6 ▸(*a*) was used to re-examine the original dataset. In every peak list, the expected Laue pattern was searched, and wherever a match was found, the pattern was indexed and the local deviatoric strain tensor extracted.

Fig. 7 ▸ summarizes the outcome. Fig. 7 ▸(*a*) shows the Laue pattern isolated by the segmentation, whereas Fig. 7 ▸(*b*) contains the experimental pattern at the center of the grain. The footprint recovered by this search [Fig. 7 ▸(*c*)] coincides with the appearance map, and additional reflections absent from the appearance map matching step are recovered during indexing. Spatial maps of the fit quality [Figs. 7 ▸(*d*)–7 ▸(*e*)] indicate that the indexing procedure is robust across the footprint. The number of indexed peaks is highest where the diffracting volume of the grain is largest [Fig. 7 ▸(*d*)], consistent with a greater illuminated volume along the beam path producing more reflections. The mean pixel deviation only increases at the margins, where the quality of the indexing result is expected to be lower because there are fewer and weaker reflections. The last panel [Fig. 7 ▸(*f*)] at the bottom reports the components of the deviatoric strain tensor. It demonstrates how to access intra-grain deformation and provides additional evidence of the consistency of the segmentation, orientation extraction and refinement workflow. The local elevated deviatoric strain values visible at the grain periphery coincide with regions of lower indexed-peak count and larger mean-pixel deviation; they are therefore attributed to reduced robustness of the strain refinement near the grain boundary.

## 3. Results and discussion

### 3.1. Statistical analysis

Given the large number of grains, a statistical summary is informative and provides further insights into the ensemble of grains present in the sample. Among all clusters returned by the segmentation, the most intense grains were selected, and the validation procedure described in the previous section was carried out for each of them. Fig. 8 ▸ shows the grain size distribution and the corresponding inverse pole figure (IPF) map of their crystallographic orientations relative to the sample surface.

The orientation distribution appears uniform, with no evident preferred orientation. Consistently, the color-coded bar plot in Fig. 8 ▸(*a*), which uses the same IPF color keys, shows no correlation between grain size and orientation, as bars of similar color are spread across the size range.

### 3.2. Materials insights

Beyond the ensemble statistics, the segmentation gives direct, grain-resolved access to information that is not readily obtained from conventional Laue analysis of such dense diffraction patterns: individual grain footprints, intra-grain strain distribution and crystallographic relationships between neighboring grains. In particular, several pairs of grains sharing similar spatial footprints but distinct indexed-peak distributions were identified as crystallographic twins (see Fig. S8).

From a materials perspective, the limited strain heterogeneity found at most grain boundaries together with the absence of preferred orientation and of any correlation between grain size and orientation points to a largely relaxed, untextured polycrystalline microstructure for this 3C-SiC wafer, a description that could not be obtained without resolving individual grains across the illuminated area.

More fundamentally, conventional indexing strategies are not designed to operate on Laue patterns of this density; the present workflow makes this kind of grain-resolved characterization possible in the first place.

### 3.3. Limitations and further developments

The principal limitation lies in the intrinsic sources of variability of the appearance maps. Different reflections have different energies, and thus different penetration depths, and result in slight differences in the edges of their appearance map. Intragranular orientation and strain gradients also contribute to this variability: as discussed in Section 2.2.1, local misorientation and elastic strain displace a reflection’s position across the grain’s footprint, which sets a practical lower bound on the LUT and tracking tolerances (more details in Section S5.1). When these gradients become large enough to rival the spacing between neighboring reflections, the situation is similar to that of overlapping peaks. Furthermore, the high spot density in each Laue diagram makes partial overlaps in the raw images likely. These overlaps may cause a spot to be absent from the initial peak list, which can later result in fragmentation of its appearance map. Both effects are observable in Fig. 5 ▸. These imperfections bias the similarity scores: when the threshold is set too strictly, a physical grain is divided into multiple clusters. Conversely, permissive thresholds risk merging partially overlapping grains. In practice, connected-component analysis and orientation-based consolidation mitigate these effects. However, it is important to conduct a thorough peak search. It may take several attempts to select the appropriate similarity thresholds. This overlap-driven limitation is shared by any peak-detection-based approach, irrespective of the downstream indexing strategy: if two reflections overlap so strongly that they cannot be resolved as separate peaks during fitting, they are absent from the peak list and cannot be processed by any method, including conventional indexing or neural-network-based approaches. When reflections remain individually resolvable but lie closer together than the LUT tolerance, they are instead assigned a single LUT entry, which can result in a peak being attributed to the wrong grain (Section S5.2, Fig. S5). A related limitation concerns very small grains, whose appearance map can reduce to only a few raster positions; in this regime, the cosine similarity becomes highly sensitive to single-pixel differences between maps which makes robust clustering difficult (see Section S5.3 for a quantitative example).

Several developments could improve the robustness and scope of the workflow. First, the construction of the look-up table can be adapted to dataset complexity. Fig. 3 ▸ shows a complex dataset for which it is difficult to assign a single nominal position to each cluster of peaks. However, for patterns with fewer peaks and clearer separation, standard clustering on detector coordinates – *e.g.k*-means, with a justified number of clusters *k*, or density-based methods such as DBSCAN or HDBSCAN – can provide centroids that directly populate the LUT, reducing reliance on a fixed proximity radius and capturing variable cluster geometry (see ROI 1 and ROI 2 in Fig. 3 ▸).

Second, when two reflections from different grains are close together on the detector, the LUT contains a single position representing both. The relative appearance map will then exhibit two regions that are recognizable as distinct grains. Extending the connected component analysis step to retain the two largest components would avoid the arbitrary loss of information on one of the two grains, provided that appropriate decision rules are applied to exclude spurious components arising from noise.

The third point regards the similarity assessment. While cosine similarity is fast and size aware, alternative metrics may better reflect specific sample conditions. Allowing user-selectable metrics, together with adaptive thresholds (discussed in Section S5 of the supplementary materials), would reduce over-segmentation.

### 3.4. Computation times

The pipeline was benchmarked on the ESRF edge computing cluster in two modes: an interactive *Jupyter* run using 40 CPU cores (total execution time ≈ 4 h) and an equivalent SLURM batch job on a 192-core node (total execution time ≈ 52 min). A breakdown of the computation times of each step of the workflow is reported in Table S1 of the supplementary materials.

These timings correspond to execution once the parameters have already been chosen; selecting them still requires dataset-specific tuning, informed by inspection of both the diffraction patterns and the intermediate results of the segmentation. This limitation, especially regarding the peak search, is shared by other methods designed to handle dense, large-scale Laue microdiffraction datasets and is discussed further in Section S2.1 of the supplementary materials.

## 4. Conclusion

This work demonstrates a robust, deterministic workflow that converts high-density Laue microdiffraction raster scans into grain-resolved representations. These representations include grain appearance maps and average diffraction patterns. A primary strength of this approach is its ability to segment these complex datasets without prior knowledge of the material’s phase or microstructure. By leveraging spatial correlations and cosine similarity clustering, the methodology successfully manages datasets containing several hundred grains, effectively bypassing the combinatorial and memory bottlenecks that hinder traditional indexing and dictionary-based methods (Micha *et al.*, 2025 ▸; Seret *et al.*, 2022 ▸).

The efficacy of this pipeline is underscored by its ability to isolate individual grain contributions where each grain contributes, on average, 60 Bragg reflections (see Fig. S7) to its respective Laue pattern. This wealth of data supports the extraction of crystallographic orientation and the refinement of deviatoric strain with a resolution of 10^−4^, all while maintaining sub-micrometre spatial resolution (Fig. 7 ▸). As shown with the polycrystalline 3C-SiC sample, these results enable comprehensive ensemble statistics on grain size and orientation distributions (see Fig. 8 ▸). This provides a quantitative view of the microstructural properties that influence the response of functional materials (Li *et al.*, 2025 ▸; Biard *et al.*, 2023 ▸; Huang *et al.*, 2025 ▸).

Unlike surface-sensitive techniques such as EBSD, this synchrotron-based polychromatic X-ray diffraction approach enables the non-destructive, *in situ* mapping of bulk polycrystalline materials. While the current study focuses on semiconductors, the methodology is readily applicable to other material classes such as metals (Imrich *et al.*, 2014 ▸), alloys (Altinkurt *et al.*, 2018 ▸; Chen *et al.*, 2016 ▸), ceramics (Magalhaes *et al.*, 2025 ▸; Ibrahim *et al.*, 2017 ▸) and oxides (Purushottam Raj Purohit *et al.*, 2024 ▸). The integration of this segmentation technique with depth-sensitive methods like differential aperture X-ray microscopy, already present on BM32, offers a clear path toward 3D mapping of grain footprints and intragranular strain fields in both synchrotron and laboratory environments.

Importantly, although synchrotron radiation provides high flux and speed, this conceptual framework for organizing complex patterns can be directly applied to laboratory-scale measurements (Whitley *et al.*, 2015 ▸; Lynch *et al.*, 2007 ▸; Zhang *et al.*, 2025 ▸).

## 5. Software availability

The analysis pipeline is implemented in Python and distributed as the open-source package *graintools*. The source code, including version history and issue tracking, is hosted on GitHub at https://github.com/BM32ESRF/graintools.

## Supplementary Material

Supporting information file. DOI: 10.1107/S1600576726007995/yr5168sup1.pdf

## Figures and Tables

**Figure 1:**
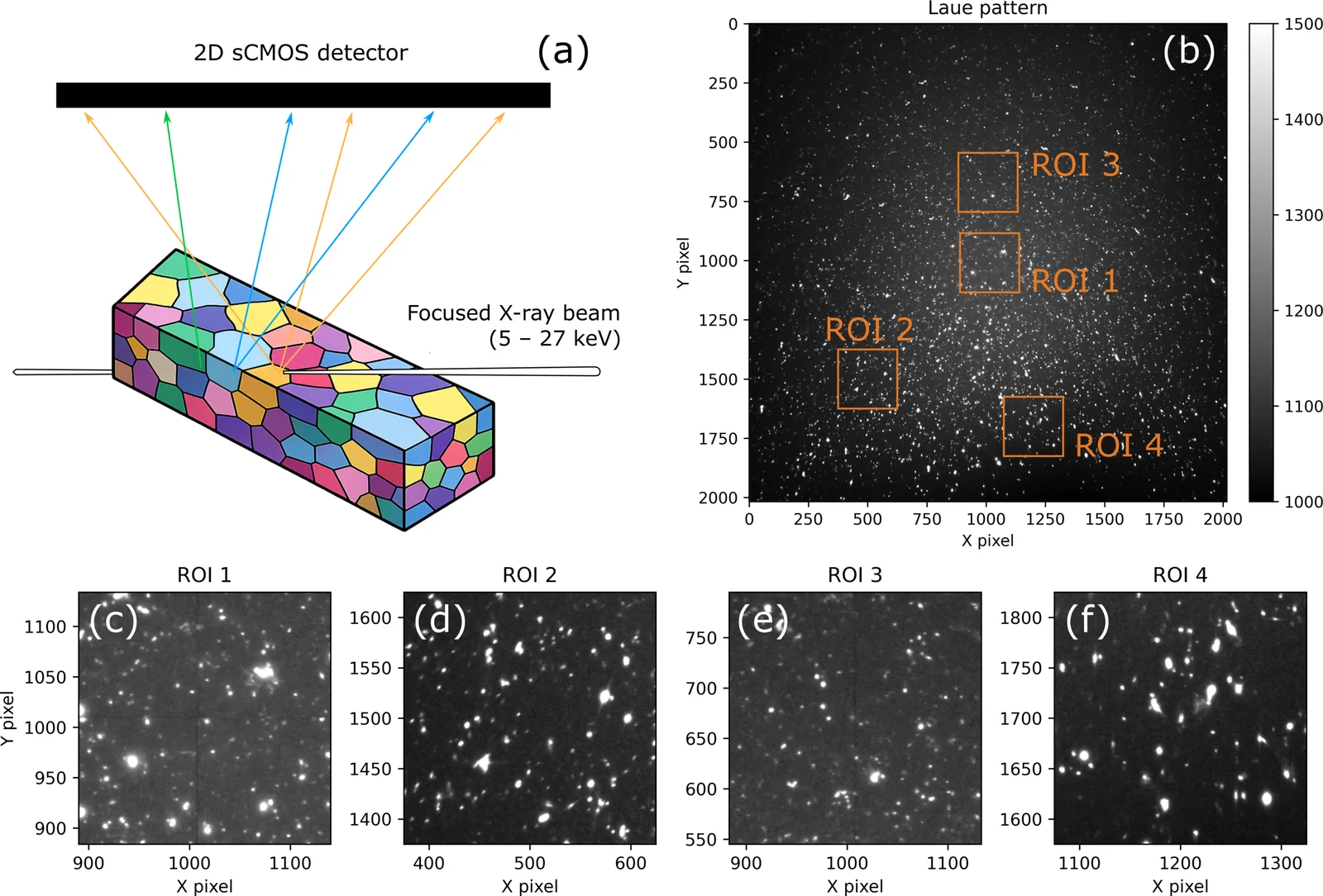
(*a*) Schematic of the diffraction geometry used at BM32 (ESRF): A focused white beam (energy range between 5 and 27 keV) illuminates a column of grains along the beam path. Multiple grains satisfy distinct Bragg conditions, and the scattered rays are collected on a 2D sCMOS detector. (*b*) Typical Laue pattern collected in a raster scan of polycrystalline SiC. The full detector (2018 × 2016 pixels) image exhibits the high spot density typical of polychromatic diffraction from a polycrystalline sample. (*c*)–(*f*) Four regions of interest (ROIs 1–4) are enlarged to illustrate local variability across the pattern: differences in spot density and intensity, variations in spot shape (from rounded to streaked or elongated), degrees of overlap, and background level. These contrasts reflect changes in local grain illumination, strain and misorientation within the illuminated volume.

**Figure 2:**
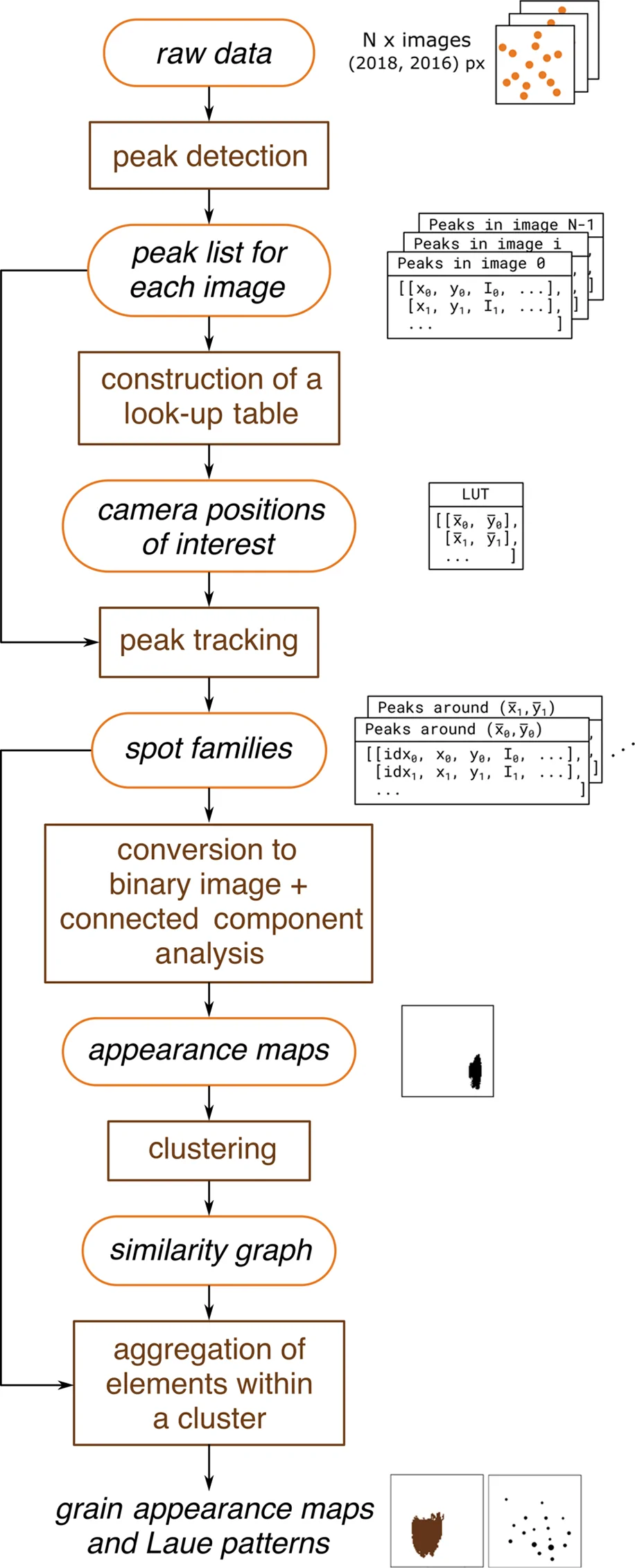
Workflow of the segmentation pipeline of Laue diffraction patterns with many grains/peaks. Brown rectangular boxes denote data-processing steps; rounded orange boxes denote the associated data objects (inputs/outputs). The thumbnails provide a visual representation of the data structures involved throughout the pipeline, which are described in the corresponding sections. The two shortcut arrows indicate data objects that are reused by a non-adjacent step further downstream, in addition to feeding the step that immediately follows them: the peak list for each image is required again during peak tracking, and the spot families are required again during aggregation of elements within a cluster.

**Figure 3:**
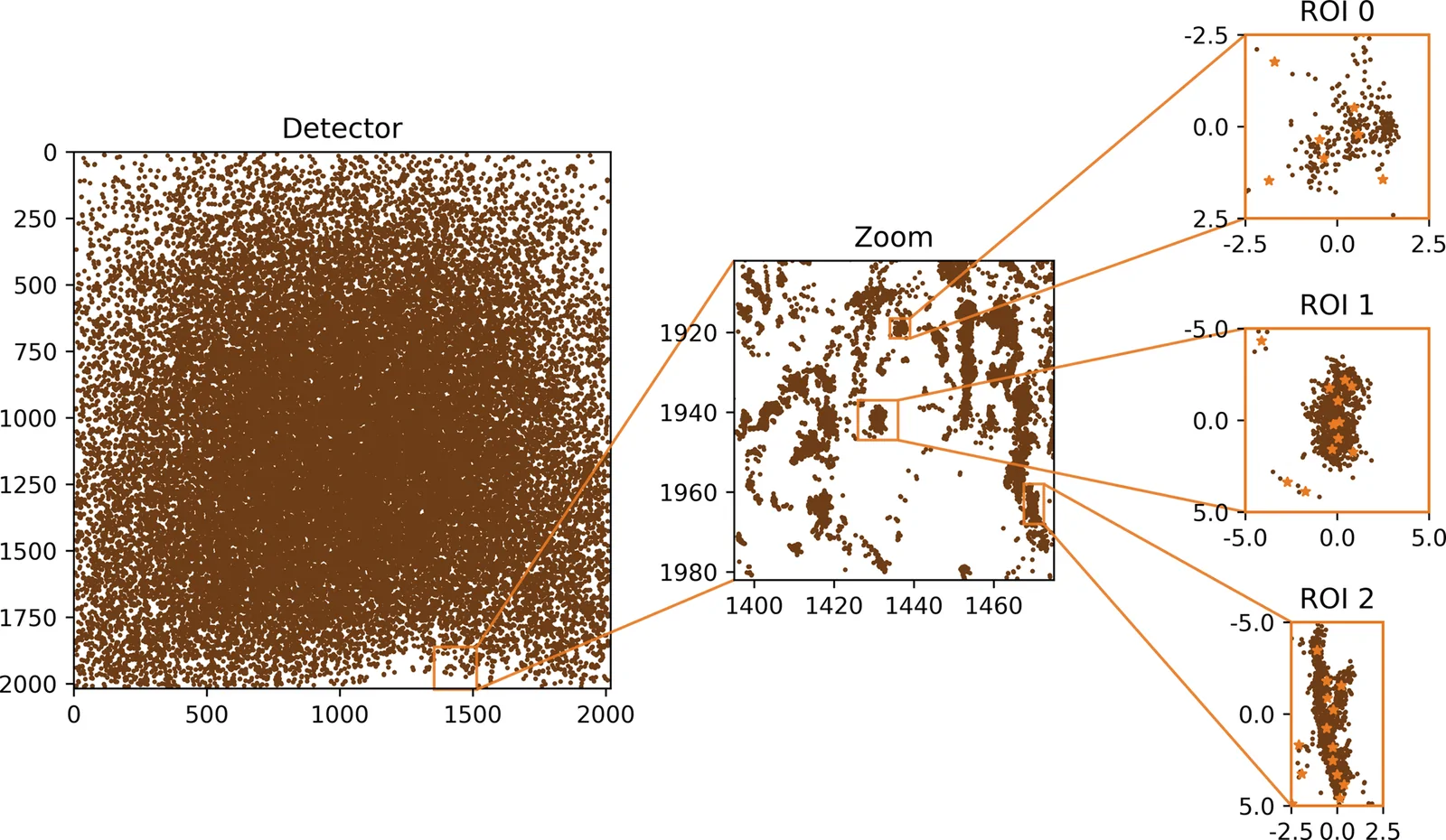
Experimental positions of all diffraction peaks on the sCMOS detector during a 101 × 101 raster scan of a 3C-SiC polycrystalline sample (representative of a 50 µm × 50 µm scan in steps of 0.5 µm in both directions). The positions of all the peaks collected on the detector during the scan are plotted on the leftmost panel. Successive magnifications reveal a specific structure in the data when moving from left to right (all axes are in pixel units). Diffraction spots organize into clusters associated with common families of lattice planes, but they can be heterogeneous in size and partially overlapping. The complexity may prevent a straightforward definition of a single nominal position for each cluster, as indicated by the orange scatter points in the three ROIs. The implications of this ambiguity and how it is dealt with are discussed in Section S5.1 of the supplementary materials.

**Figure 4:**
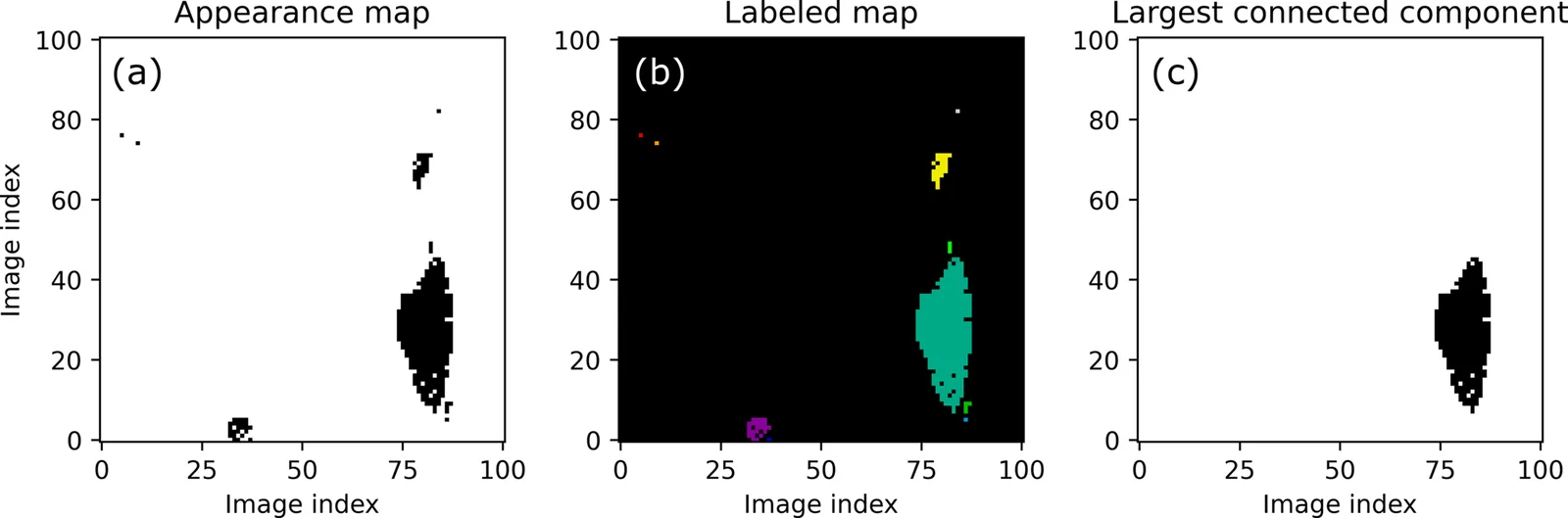
A spot family contains images in which a reflection appears across the raster scan. This information can be converted into a two-dimensional diagram (*a*), called an ‘appearance map’, which shows where the reflection appears on the sample surface. These maps feature the shape of a grain and possibly spurious regions that do not belong to it. To remove them, CCA is used. The output of CCA is a labeled image (*b*) that identifies the different connected components, indicated here by colors corresponding to different labels. (*c*) Only the largest among them is kept to isolate the grain of interest.

**Figure 5:**
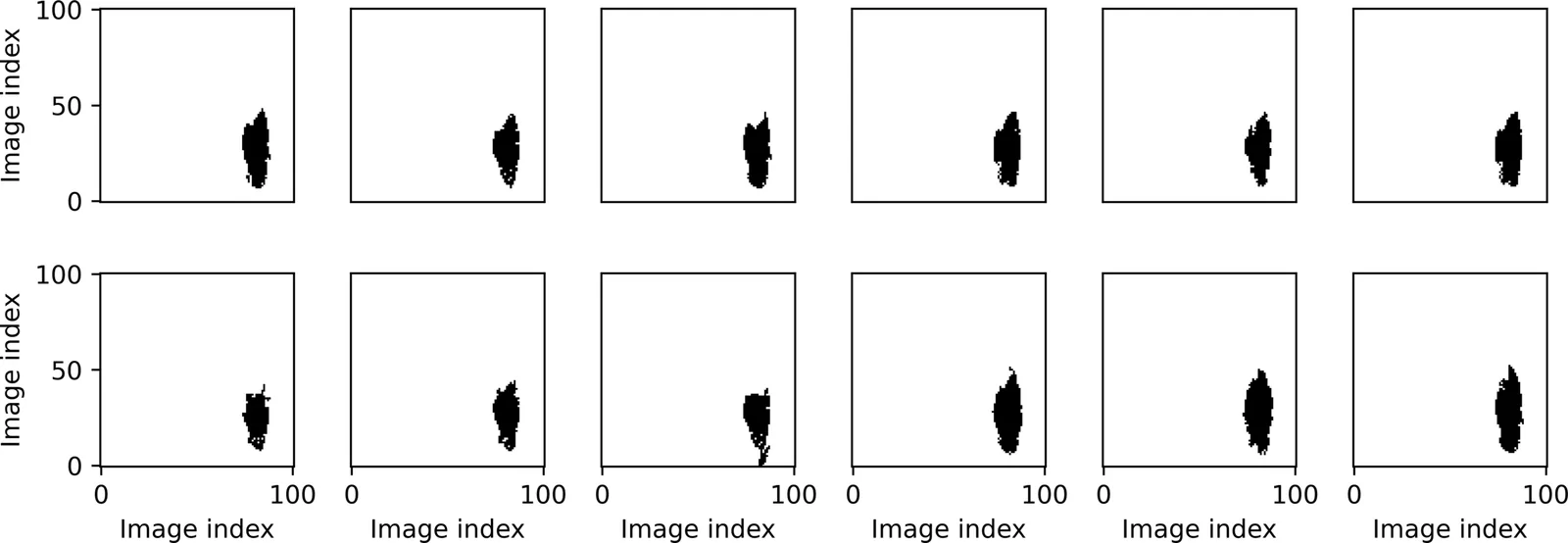
Example of some elements of a cluster that were calculated with a cosine similarity threshold of 0.85. The total cluster size is 75 matches.

**Figure 6:**
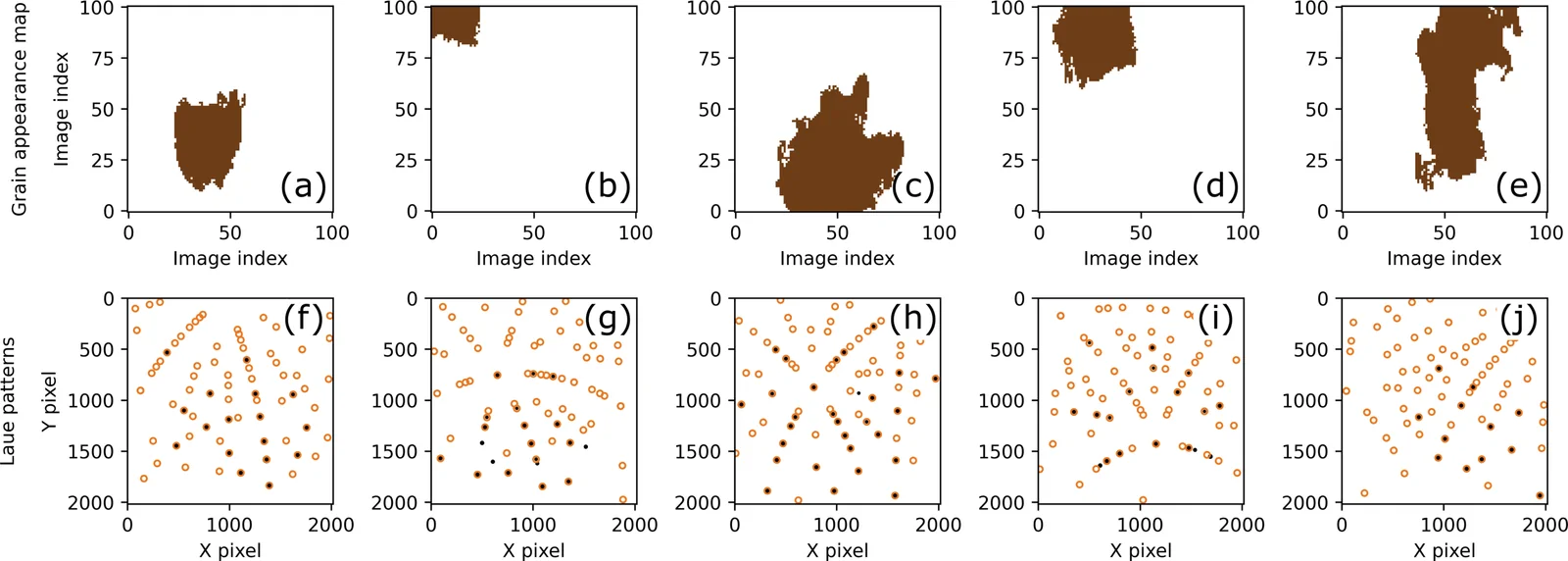
Examples of the segmentation output after having extracted the crystal orientation. The first line shows the appearance maps of the crystal grains. The corresponding diffraction patterns are shown below. The measured (simulated) patterns are in black (orange).

**Figure 7:**
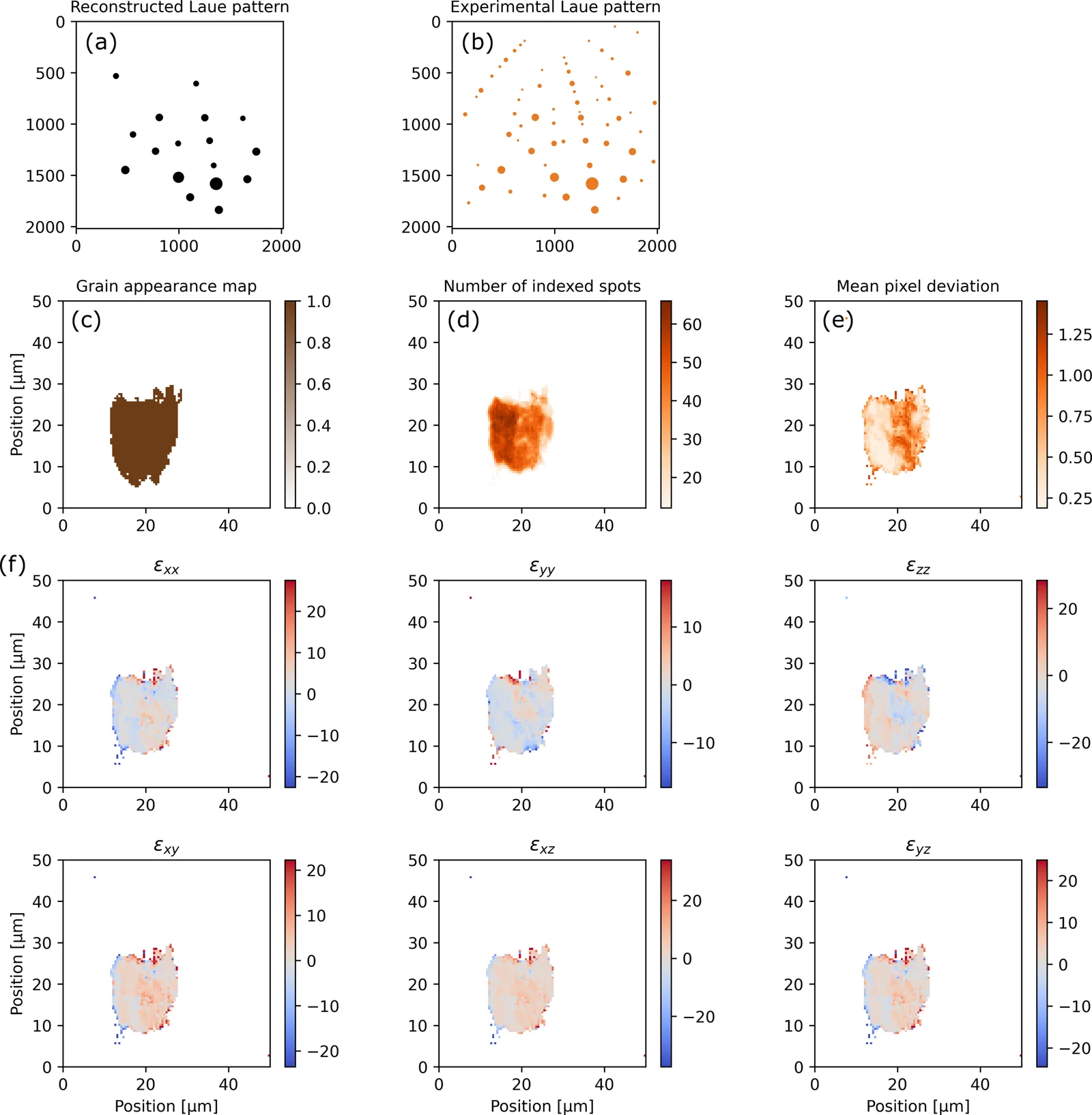
Result of the validation process for one grain. (*a*) Laue pattern isolated by the appearance map segmentation. (*b*) Laue pattern, corresponding to the same orientation, found at the center of the grain. (*c*) Binary image representing the grain shape as determined by the segmentation. The following panels illustrate the information obtained by indexing the pattern in the dataset, given the orientation. (*d*) Map of the number of indexed peaks. (*e*) Mean deviation between the experimental and theoretical peak positions, in pixel units. (*f*) Deviatoric strain map. Each panel reports the values of the deviatoric strain tensor components. The color bar shows the range of mean ± 3 standard deviations calculated for each deviatoric strain component, all in units of 10^−4^. The (*x*, *y*, *z*) axes are in the crystallographic frame, *i.e.* along the crystallographic axes *a*, *b* and *c*, respectively.

**Figure 8:**
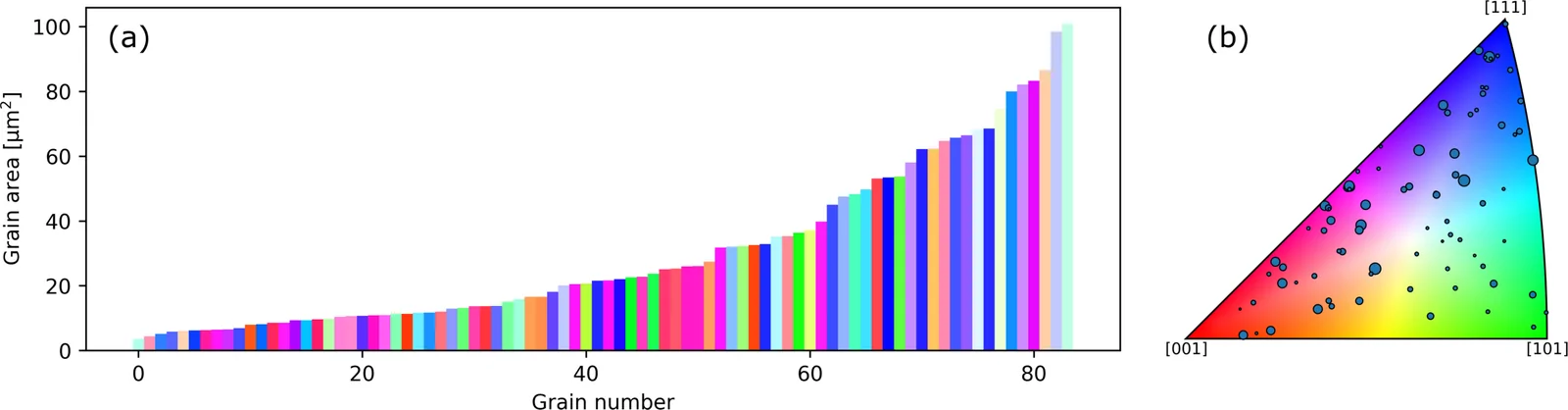
Statistical analysis of the grains. (*a*) Bar plot of the grain sizes for the 84 segmented grains. (*b*) IPF map of the grain orientations with respect to the direction normal to the sample surface. Colors in the bar plot (*a*) reproduce the IPF key in (*b*) and marker sizes reflect relative grain sizes.

## Data Availability

The raw data and *Jupyter* notebooks supporting the results of this article are openly available from Zenodo at https://zenodo.org/records/18618617. The *graintools* analysis software is openly available from GitHub at https://github.com/BM32ESRF/graintools.
