# Supplementary material for: Deterministic segmentation of grains in dense Laue microdiffraction datasets
Source: J Appl Crystallogr. 2026 Sep 25;59(Pt 5):1584–94. doi: 10.1107/S1600576726007995 (PMC13636047; doi:10.1107/S1600576726007995)

JOURNAL OF APPLIED CRYSTALLOGRAPHY

Volume 59 (2026)

Supporting information for article:

Deterministic segmentation of grains in dense Laue microdiffraction datasets

Sergio Bongiorno, Jean-Sébastien Micha, Samuel Tardif and Joel Eymery

Supplementary Materials

Deterministic segmentation of grains in dense Laue microdiffraction datasets

Sergio Bongiorno[1], Jean-Sébastien Micha[2], Samuel Tardif[1] and Joël Eymery[1]
[1] Univ. Grenoble Alpes, CEA, IRIG, MEM, NRX, 38000 Grenoble Cedex, France
[2] Univ. Grenoble Alpes, CNRS, UMR SyMMES, CEA-Grenoble, IRIG, 38000 Grenoble, France

The supplementary materials expand on the following topics:

1. *Software and resources*
2. *Computational resources and time*
3. *How to implement a correct PeakSearch*
4. *Look-up table with the experimental data*
5. *Peak tracking: choice of the tolerance*
6. *Impact of connected component analysis on appearance map matching*
7. *The grain refinement procedure*
8. *Validation on synthetic dataset with known ground truth*

S1. Software and resources

The analysis pipeline is implemented in Python and distributed as the package **graintools**. The software is openly available and can be installed from the Python Package Index (PyPI) using `pip`. The full source code, including version history and issue tracking, is hosted on GitHub at:

https://github.com/BM32ESRF/graintools

The package is organized into the following submodules:

```
graintools
├─ utils          utility functions used in other submodules
├─ peaks          Operations on peak lists (e.g., union, intersection…)
├─ simulation     tools to generate synthetic Laue microdiffraction datasets
└─ segmentation
    ├─ lut.py          Construction of the look-up table
    ├─ tracking.py     Identification of spot families
    ├─ amaps.py        Appearance maps and connected component analysis
    ├─ matching.py     Appearance map matching
    └─ result.py       Structuring of the result and file input/output
```

An example workflow is provided in the repository's examples directory. The associated Jupyter notebook demonstrates the construction of a minimal yet representative synthetic dataset and illustrates how to use the segmentation pipeline in this given case.

S1.1 Raw data and Jupyter notebooks

Link to Zenodo

S2. Computational resources and time

The computational cost of the segmentation pipeline was evaluated by executing the same workflow in two different operating modes on the ESRF edge computing infrastructure. In the first case, the pipeline was run from a Jupyter notebook using 40 CPU cores on a SLURM-managed compute node. This configuration corresponds to the interactive environment routinely used during method development, parameter tuning, and exploratory analysis. In the second case, the identical workflow was submitted as a batch SLURM job to a compute node with 192 CPU cores (AMD EPYC 9654, Genoa), representative of the execution once all parameters have been fixed.

Table S1 reports the time required by each major step of the segmentation pipeline for both execution modes. Jupyter-based execution plays a central role in the development process because it provides the flexibility needed to refine parameters iteratively and assess intermediate results. The SLURM batch execution, by contrast, reflects the final deployment of the pipeline on large datasets, where interactivity is no longer required, and computational throughput becomes the primary concern.

Computation time required by the segmentation		
	Resources used	
Data treatment step	40 cores (Jupyter)	192 cores (SLURM job)
PeakSearch	77 m 28 s	10 m 38 s
LUT construction / clustering	6 m 4 s	2 m 32 s
Peak tracking	74 m 39 s	16 m 25 s
Appearance maps + CCA	1 m 27 s	0 m 40 s
Appearance map matching	81 m 54 s	22 m 33 s
Total	4 h 0 m 32 s	52 m 48 s

Table S1. Breakdown of the computation times required by each step of the segmentation. The pipeline ran on ESRF's edge computing system. It was run on a 40-core Jupyter notebook and sent as a 192-core SLURM job.

S2.1 Parameter tuning and its time cost

The timings in Table S1 reflect the cost of running the pipeline once its parameters – peak-search settings, LUT and tracking tolerances, similarity threshold — have already been fixed. Selecting these parameters is, at present, a manual step rather than an automated one. Peak search in particular requires some expert insight into the morphology of the diffraction spots in a given dataset, which cannot currently be inferred automatically; the only configuration where this is not an issue is the trivial case of an isolated single crystal, for which manual tuning is in any case inexpensive.

This is not a shortcoming specific to the present method: any software intended to process more than a few thousand Laue patterns faces the same constraint. While a single peak search takes only a few seconds, the cost of analyzing an entire raster scan scales rapidly with dataset size, so that wrapping an automated parameter search around the core computation introduces an overhead that is difficult to justify for the datasets targeted here. CPU parallelization across compute clusters mitigates this cost, as shown in Table S1, but such infrastructure cannot be assumed to be routinely available to all users.

The same considerations apply to the segmentation parameters, especially for a dataset as dense as the one analyzed in this work. For smaller or simpler datasets, however, automated parameter selection becomes far more tractable, since the processing time per step is short enough to allow iterative or exploratory tuning. The simulated dataset described in Section S4 illustrates this: although it comprises more than one million peaks in total, the full segmentation pipeline runs in under a minute. Promising directions for automation already exist — for instance, the similarity-threshold metric discussed in Section S5.3 — and as the workflow is applied to a broader range of samples, the resulting experience is expected to support a more automated pipeline for cases where dataset complexity allows it.

S3. Implementing a correct PeakSearch

Peak search is the first step in the workflow. The following assumes that each image has been fully exploited. This means extracting as many peaks as possible while ensuring poor fits are not included in the list. In dense Laue patterns, a single parameter set is rarely sufficient because spot intensity and size vary widely and overlap is common. For this reason, we run three rounds of peak searching and then merge the results.
We use the PeakSearch routine from LaueTools, which detects candidate local maxima and refines their positions and shapes by fitting the 2D intensity profile within a square Region of Interest (ROI) to a Gaussian. Three key parameters define each round."

- *Intensity threshold*: minimum pixel intensity for a local maximum to be considered as a candidate peak.

- *Box size*: the linear half-side (in pixels) of the square ROI centered on the candidate maximum used for the fit. Larger boxes better capture wide peaks but increase the chance of including nearby peaks and background gradients.
- *Pixel deviation*: the maximum allowed displacement (in pixels) between the initial local-maximum position and the refined fitted peak position. It acts as a limit on how far the fit is allowed to "walk."

The three rounds used here are:

Round	Intensity threshold	box size	pixel deviation
#1	4000	20	10
#2	1000	15	6
#3	150	7	0.7

where the intensity thresholds are intended above the background.
The logic behind this choice is that very intense peaks are often large (and can be slightly saturated, asymmetric, or affected by local overlap), so we fit them with a larger box to include the full spot footprint, and we allow a large pixel deviation because the initial local-maximum estimate can be imprecise (e.g., plateaued maxima or skewed intensity). As we move to weaker peaks, we expect them to be smaller and more localized. Therefore, we reduce the box size to avoid swallowing neighboring intensity, and we become more demanding by tightening the allowed pixel deviation—weak peaks should not require the fit to drift significantly to be explained, and large drifts are usually a sign of a spurious detection or a misfit.
After the three passes, detections are consolidated (removing duplicates within a small proximity tolerance) to form a single peak list per image suitable for the subsequent steps.
Figure S1 illustrates why this multi-round strategy is necessary. It compares the outcome of a single-pass peak search to a multi-round procedure on two ROIs. The multi-round procedure captures both large, intense peaks and small, weak spots with fewer incorrect fits.

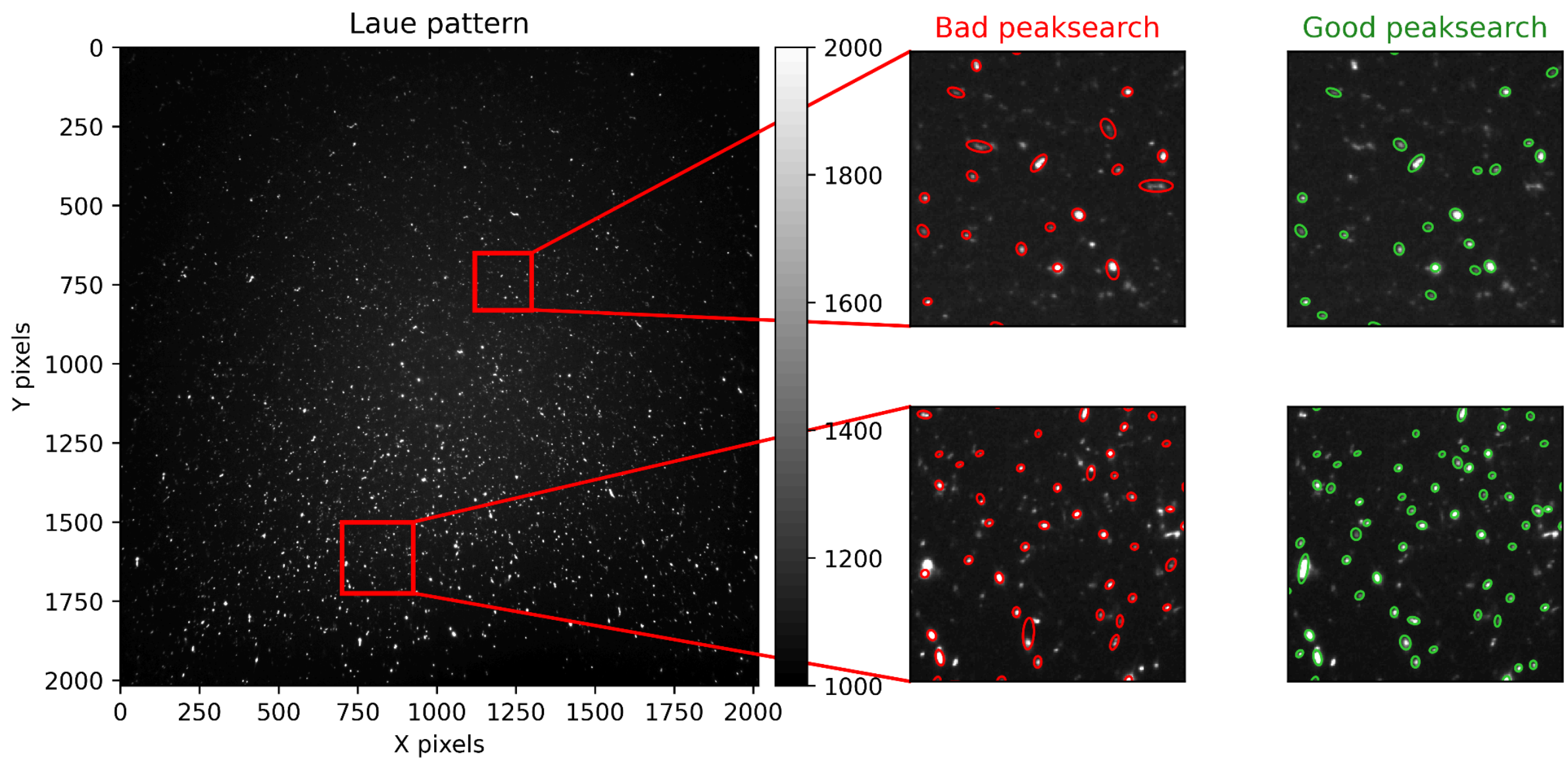

Figure S1. Importance of a multi-round peak search in dense Laue patterns. (left) Representative Laue detector image; two regions of interest (ROIs) are highlighted and enlarged on the right (top and bottom rows). (right) Peaksearch results overlaid on each ROI. A single-pass peaksearch, in red, uses one parameter set and therefore fails to handle the coexistence of large/intense and small/weak spots, leading to missed detections and/or incorrect fits. A multi-round strategy, whose results are plotted in green, combines passes tuned to different intensity/size regimes, capturing more peaks with more accurate fits across spot morphologies.

S4. Look-up table

In this section — and in the following one — whenever the key ideas of the segmentation become hard to visualize due to the complexity of the real experimental dataset, it is useful to refer to a simulated dataset. Using a simulation makes the workflow easier to visualize and discuss: the ground truth (grain positions and sizes, and peak origins) is known, and the complexity can be controlled. While simpler, the simulated dataset remains representative of the essential features and pitfalls that matter for the method, such as grain morphology, dense peak clusters, and overlapping reflections. Section S4.1 briefly describes the simulation and the dataset generated.

S4.1 Simulating a dataset

To keep the discussion close to the experimental conditions, a synthetic dataset is generated with the same spatial extent and sampling as the real scan. A 50 µm × 50 µm surface is raster scanned with a 0.5 µm step in both the *x* and *y* directions, yielding 10201

(101×101) positions. The surface is completely covered with grains. To make implementation easier, grains are constructed with comparable areas and randomized boundaries. Users can further increase realism by using larger sizes and heterogeneous morphologies. In this configuration, the tiling contains 169 grains, each assigned an

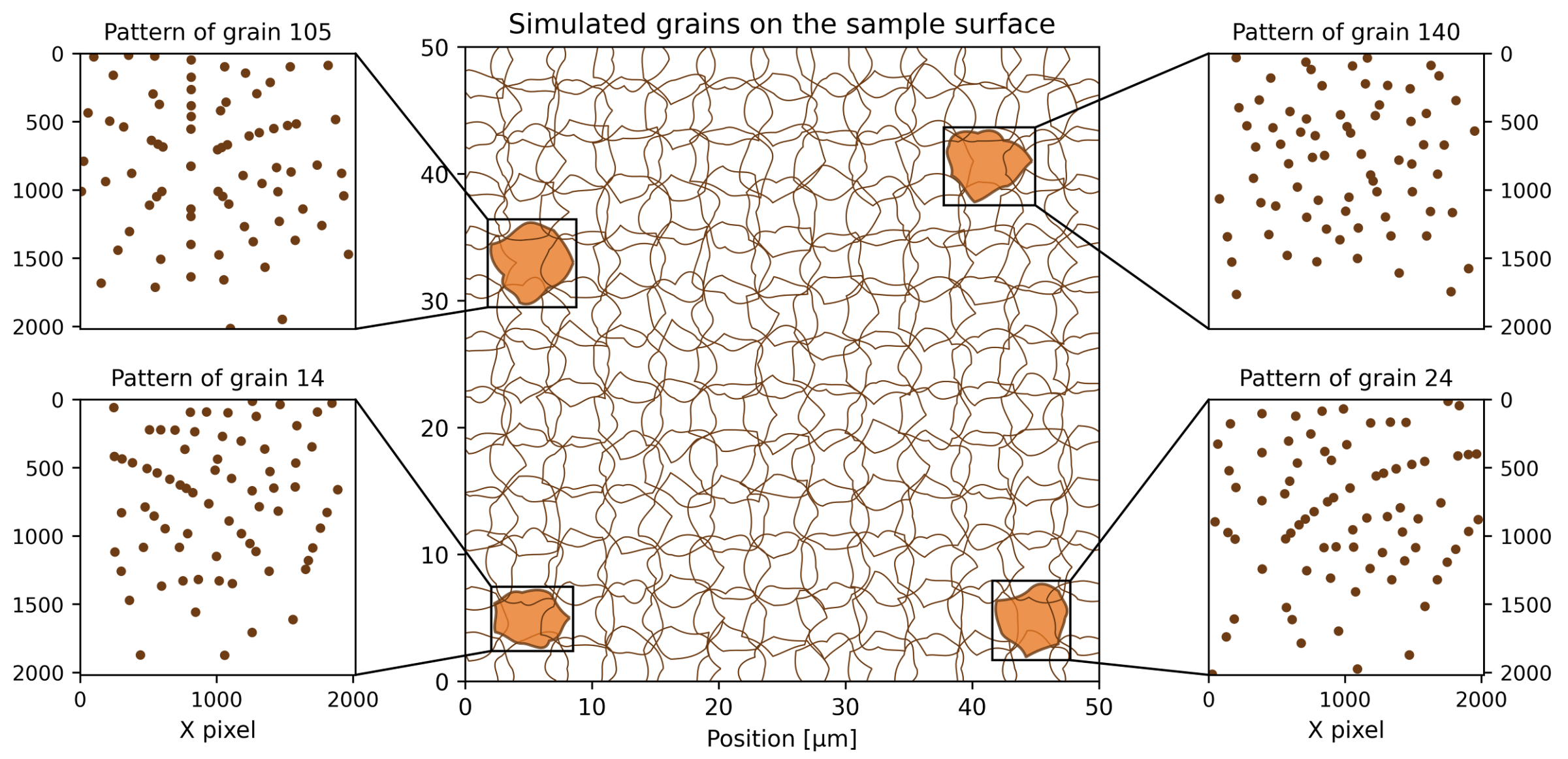

Figure S2. Synthetic microstructure used to generate the simulated scan: a 50 µm × 50 µm surface is tessellated into 169 grains with randomized boundaries and assigned random orientations. Insets show examples of the simulated Laue patterns produced by individual grains.

independent random crystallographic orientation. The resulting synthetic microstructure and examples of the corresponding single-grain Laue patterns are shown in Fig. S2.

For each raster position, the number of grains containing the point is evaluated, and a simulated Laue pattern is generated for each contributing grain using its assigned orientation, with an additional small orientation jitter introduced by adding noise with amplitude 0.01° to each Euler angle.

This procedure yields 10201 peak-lists, each corresponding to a detector pattern containing one or more superimposed Laue patterns depending on whether the scan position lies fully inside a grain or in an overlap region.

A detector-space view of the full simulated dataset is obtained by pooling together all peak positions from the 10201 peak lists and plotting them in a common detector reference frame (Fig. S3). As in Fig. 3 of the main text, this reveals that peak observations do not fill the detector uniformly but organize into compact clouds around recurrent coordinates, corresponding to reflections that reappear across the raster. In the simulated case, these clusters are much easier to distinguish. The simplified microstructure contains a single layer of grains, which reduces superposition and limits heterogeneity in peak morphology and overlaps. The zoomed regions in Fig. S3 illustrate this structure, displaying well-separated clusters of different shapes and spreads. This is precisely the information targeted by the look-up table (LUT). Each cluster can be represented by one or a few nominal detector positions, which later serve as tracking anchors.

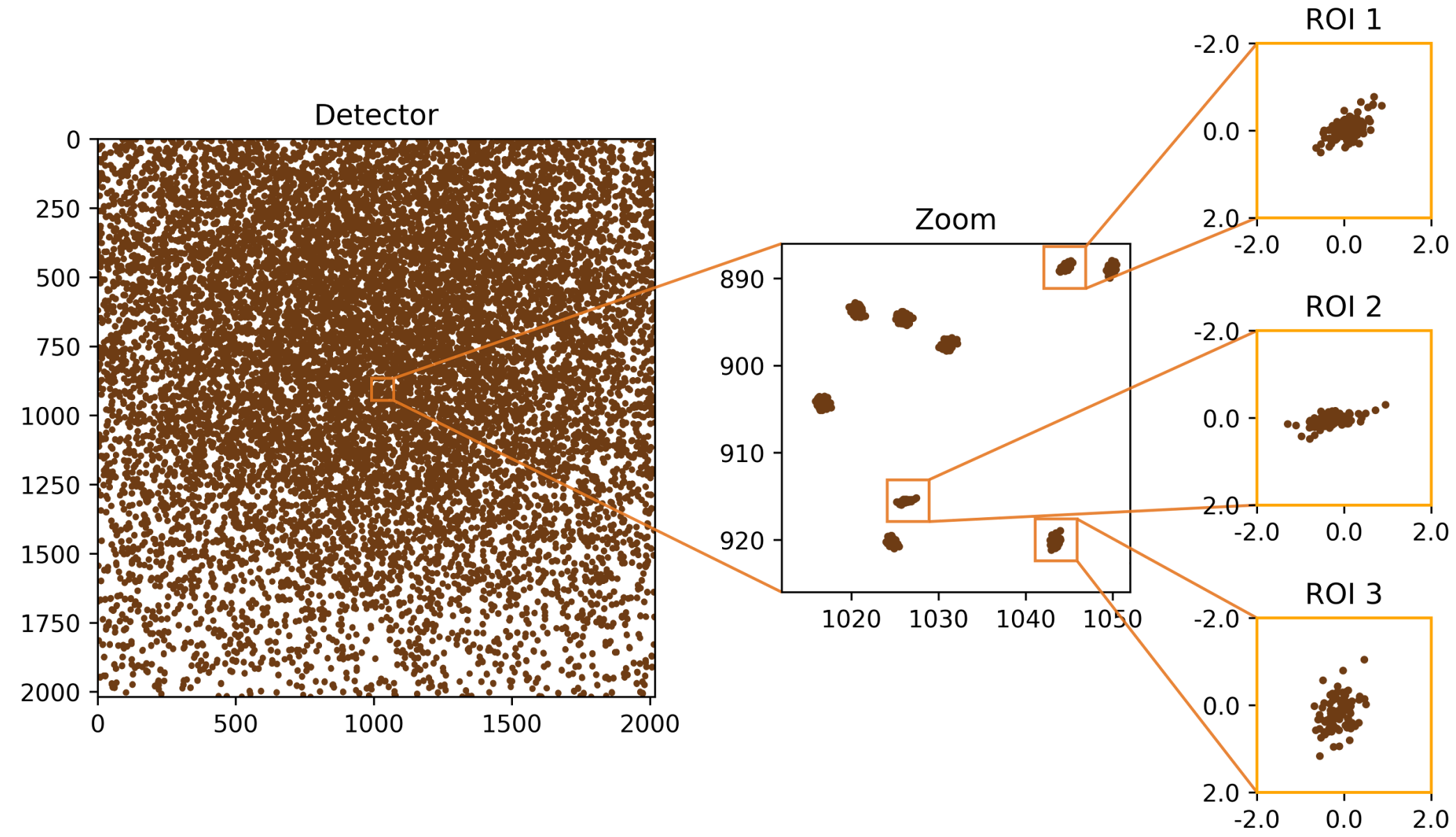

Figure S3. A 101x101 raster scan (representative of a 50μm x 50μm scan in steps of 0.5 μm in both directions) of a SiC polycrystal was simulated, comprising 169 grains. The positions of all the peaks collected on the detector during the scan are plotted on the leftmost panel. A zoomed detector region (center) highlights the clustered structure, and three representative regions of interest (ROI 1–3, right) show individual peak clouds at higher magnification. Compared with the experimental counterpart (Fig. 3 in the main text), clusters are more clearly resolved here because the simulated microstructure contains a single grain layer. These recurrent peak clouds are the features later encoded in the look-up table through representative detector coordinates (e.g., cluster centroids).

Because the clustering structure is comparatively clean in this simulated dataset, standard clustering techniques (e.g., density-based methods or mixture models) could be used directly in detector space to estimate cluster centroids, which would then populate the LUT. This strategy is generally not viable for the experimental dataset shown in Fig. 3 of the main text, where the much higher peak density, stronger overlap between neighboring clusters, and broader variability in spot shape make centroid assignment ambiguous and highly sensitive to algorithmic choices. In that regime, constructing the LUT through a physically motivated reduction of redundant peak observations provides a practical way to handle dataset size while preserving a direct interpretation in terms of recurring reflections.

S4.2 Description of the algorithm

The look-up table is built by taking advantage of a basic redundancy in Laue scans: across thousands of patterns, many reflections reappear at nearly the same detector coordinates. If all peak positions from the dataset are plotted together, these repeated reflections form dense clusters. The purpose of the LUT is to replace the full (and highly redundant) collection of peak positions with a much smaller set of representative detector coordinates that can later be used as anchors during segmentation.
To keep the computation manageable, the dataset is processed in chunks (Fig. S4). When the scan grid shape (nrows, ncols) is unknown, images are grouped into consecutive blocks

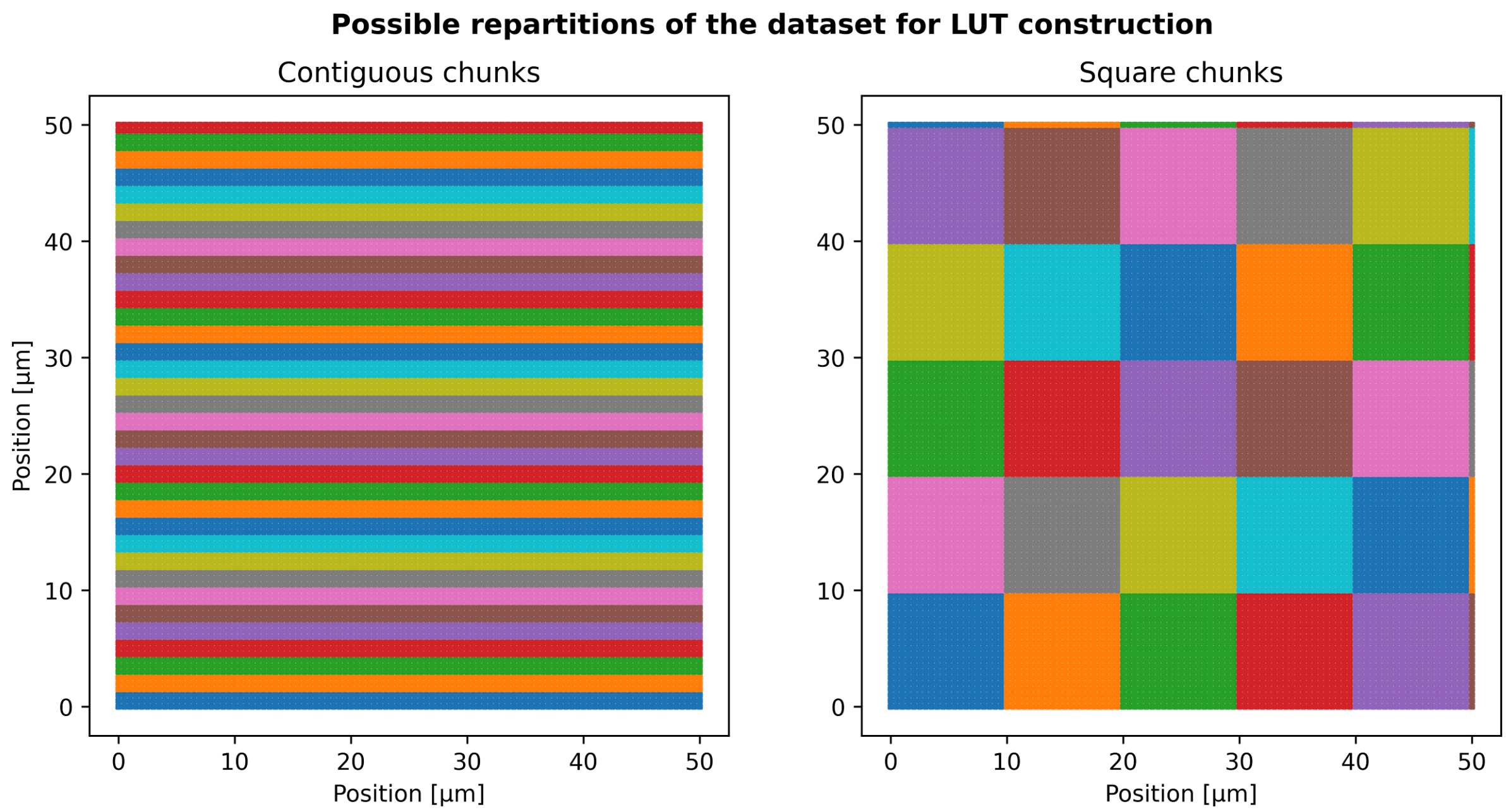

Figure S4. Schematic illustration of how the dataset is partitioned before processing. If the scan shape is not provided, images are grouped into consecutive blocks (linear chunking, left). If the scan shape (nrows, ncols) is known, images are grouped into square sub-grids (grid chunking, right), which preserves spatial locality in the raster scan and typically groups together patterns with more similar peak content.

("linear" chunking). When the shape is known, the dataset is instead split into square sub-grids, which is closer to the physical organization of the scan because neighboring points sample nearby regions and often share similar peak content. Within each chunk, all peak lists are concatenated, and peaks closer than a chosen distance tolerance are merged by keeping their average position (see Figure S5a). This step removes duplicates inside the chunk and yields a compact list of "unique" peak coordinates for that portion of the dataset.
The chunk results are then merged into a global LUT. Each peak coordinate produced by a new chunk is compared to the current LUT: if no existing LUT entry lies within the same tolerance, the peak is added; otherwise, it is already represented and is ignored. Nearest-neighbor searches are used so that this comparison stays fast as the LUT grows. Because chunks are independent, they can be processed in parallel, while the final merge only involves the much smaller chunk-level summaries. The output is an array of detector coordinates—one per recurrent peak cluster at the selected tolerance—that provides a physically meaningful compression of the dataset and serves as the LUT for the subsequent steps.

S5. Choosing the tolerance values

The method relies on three tolerance parameters that should be chosen with care because they control three different operations: (i) the tolerance used to build the look-up table (LUT) by merging repeated peak observations into representative detector coordinates, (ii) the tolerance used for peak tracking around each LUT coordinate to assemble spot families, and (iii) the tolerance (or similarity threshold) used later to group similar appearance maps.

These parameters are related, but they do not play the same role, and using a single value for all three generally leads to sub-optimal behaviour.

The parameters chosen for the simulation are reported below

**Pipeline step**	**tolerance value**
LUT construction	1.5 [px]
Peak tracking	2.5 [px]
Cosine similarity	0.80

S5.1 LUT tolerance

The effect of the detector-space tolerances is illustrated in Fig. S5 using peak clusters from the simulated dataset. The first row shows the LUT construction step: each peak cloud is reduced to a single representative position by removing duplicates within a circular neighborhood (blue marker and dashed circle). In practice, the LUT tolerance is chosen by inspection by zooming into the detector-space peak clouds (Fig. S3) and estimating a typical cluster extent. A single "perfect" tolerance rarely exists, since cluster sizes vary substantially across the detector; consequently, some reflections are expected to be represented by multiple LUT entries, especially in the experimental dataset, where peak clusters are broader and more heterogeneous (Fig. 3 in the main text).
Splitting one physical cluster into two LUT positions inevitably introduces a small level of redundancy: after peak tracking (typically performed with a slightly larger tolerance than LUT construction), both LUT positions may track the same underlying reflection and therefore yield two spot families carrying essentially the same information (and, in turn, the same appearance map). This redundancy is benign because the subsequent similarity clustering groups these duplicated families together, and the final pattern reconstruction simply contains a duplicated spot that can be filtered out when assembling the final peak list.

S5.2 Peak tracking tolerance

The second row of Fig. S5 shows the tracking step: peaks are searched in each image within a tolerance window around the LUT position (red star); in practice, a square window is used for speed (dashed red square). Peak tracking is typically performed with a slightly larger tolerance than LUT construction, so that moderately spread clusters are still recovered as a coherent spot family. Figure S5 clarifies the associated trade-off: increasing the tracking tolerance improves recall for broad clusters, but can temporarily mix nearby reflections.
This mixing can lead to non-ideal intermediate appearance maps. In the "two-cluster" case (middle column), the neighboring cluster lies entirely within the tracking tolerance, tracking collects peaks from two distinct reflections, and the appearance map contains two well-defined grain regions. After connected-component analysis, only the largest region would be retained (here the top-right grain), which we know to be incorrect from the ground

truth. As a consequence, a smaller grain loses one of its peaks and the larger one gains a peak that does not belong to its pattern. In the “partially captured” case (right column), the neighboring cluster is only partly recovered during tracking, so the appearance map contains a dominant grain and a secondary region with missing pixels; connected-component filtering removes this fragmented region, and the peak remains correctly assigned.

Importantly, neighboring clusters also have their own entries in the LUT. In the “two-cluster” configuration, the appearance map associated with the neighboring LUT entry mirrors the situation in Fig. S5h, and connected-component analysis removes the incorrect region so

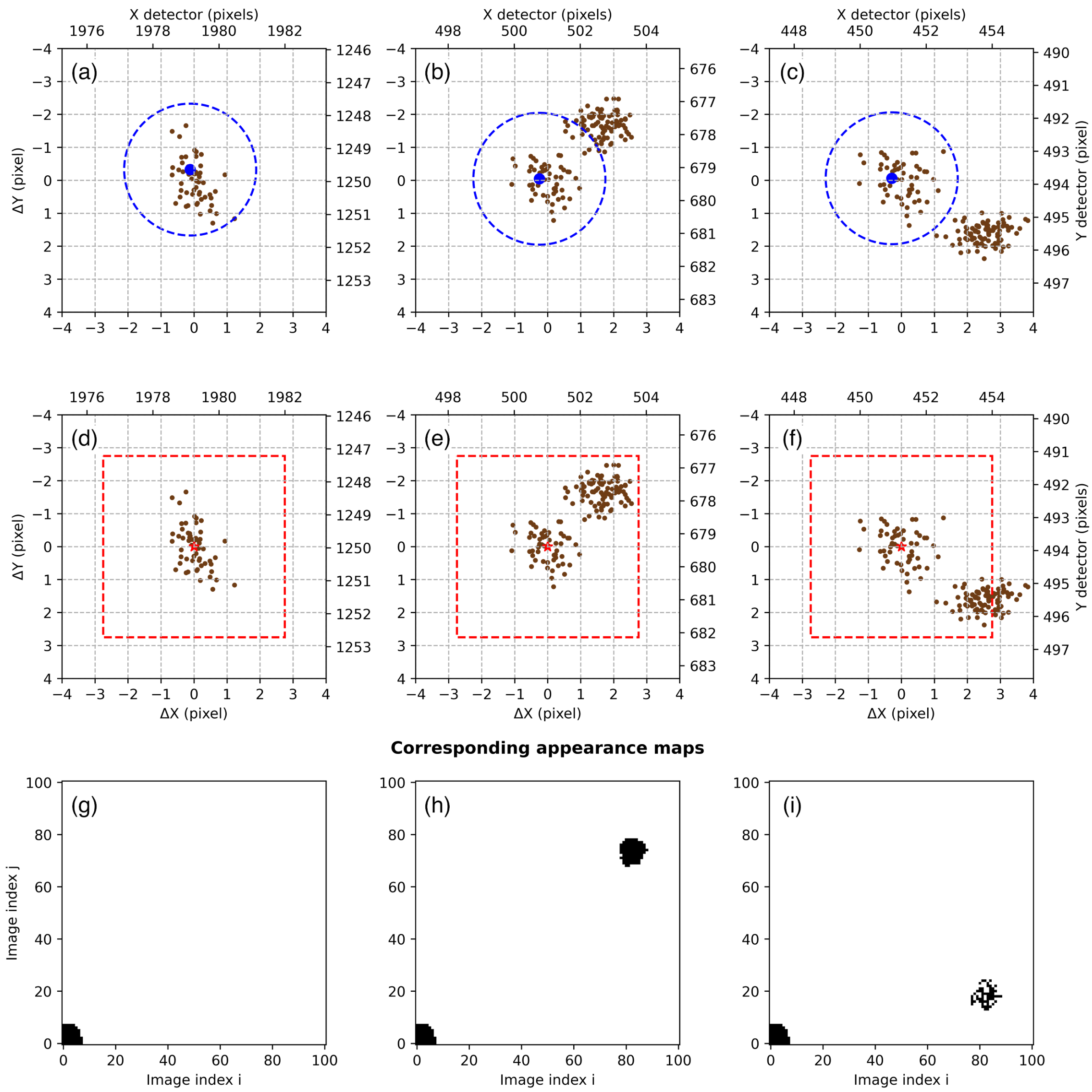

Figure S5. Schematic examples built from the simulated dataset (panels a–i). Columns illustrate three configurations: an isolated peak cluster, two clusters closer than the tolerance, and two clusters slightly farther apart. Top row: LUT construction replaces each cluster by one representative detector coordinate by merging peak observations within a circular tolerance (blue marker; dashed circle). Middle row: peak tracking around the LUT position (red star) searches within a square tolerance window for speed (dashed red square). Bottom row: resulting appearance maps. When a second cluster falls entirely within tolerance, tracking mixes reflections and yields an appearance map containing two grain regions; when the second cluster is only partially captured, the spurious region becomes fragmented. Connected-component analysis retains only the dominant connected region, which can lead to incorrect assignment in the fully-overlapping case but filters out the fragmented spurious region in the partial-overlap case.

that peaks are ultimately assigned to the correct grain. In the “partially captured” configuration, the neighboring LUT entry produces the complementary incomplete region (opposite to Fig. S5i), which is again removed by connected-component filtering, leading to a correct final assignment.

S5.3 Similarity threshold: appearance map matching

The impact of connected component analysis (CCA) on appearance map matching was quantified using the appearance maps presented in Figures 4 and 5 of the main text. This analysis is shown for a single representative appearance map from the experimental dataset, because experimental appearance maps exhibit a variability and complexity that the simulation cannot fully reproduce. The matching procedure was performed as a function of the similarity threshold in two configurations: using the raw appearance maps, and using only the largest connected component extracted from each map after CCA.

Figure S6 shows the number of matches obtained as a function of the similarity threshold for both cases. For all threshold values, the number of matches obtained after CCA is higher than when using raw appearance maps. The difference is particularly significant when similarity thresholds exceed approximately 0.80. In other words, it is significant when the metric effectively probes meaningful similarity rather than loose correlation.

For high threshold values, CCA leads to a marked increase in the number of matches. This behavior is attributed to the removal of isolated features in the appearance maps, which originate from spurious diffraction spots and reduce similarity scores. By restricting the comparison to the largest connected component, the matching focuses on physically meaningful regions, thereby enhancing the discriminative power of the similarity metric.

Both curves exhibit a comparable roll-off at high threshold values, suggesting that the decay of the number of matches could be used to identify an optimal similarity threshold for a given appearance map. While such an adaptive strategy could be advantageous, it is computationally impractical for the present dataset. Evaluating matches for a single threshold is computationally expensive, and extending this procedure to multiple threshold values for each appearance map would be impractical.

The simulated dataset is not used for this quantification because it contains only a single grain layer and does not include intensity/energy-dependent effects, e.g., varying penetration depths, which contribute significantly to the structure of experimental appearance maps

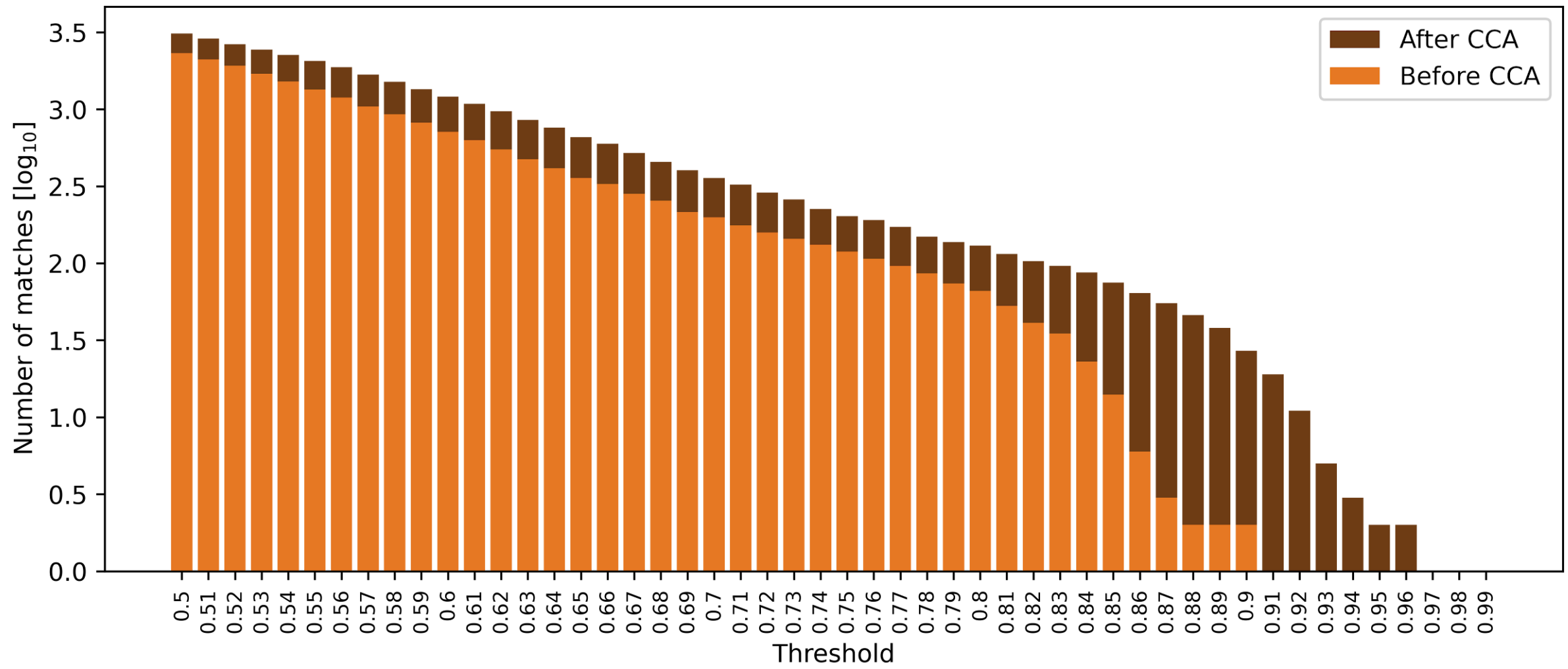

Figure S6. Number of appearance map matches as a function of the similarity threshold, computed with and without connected component analysis (CCA). Matching performed on raw appearance maps (before CCA) is compared to matching restricted to the largest connected component of each map (after CCA). The curves are obtained for a representative appearance map from the experimental dataset, since experimental maps exhibit variability not captured by the simulation (single grain layer and absence of intensity/energy-dependent effects). While CCA increases the number of matches for all thresholds, the effect becomes pronounced for similarity values above ~0.80, where isolated features are suppressed, and similarity is dominated by physically meaningful regions.

S6. Grain refinement

Because peak assignment is not guaranteed to be perfect at the segmentation stage, each segmented Laue pattern is validated by indexation. Indexing provides an immediate physical consistency check: peaks that do not belong to the pattern are naturally rejected during the indexing procedure, whereas correctly assigned reflections reinforce a stable orientation solution. Beyond validation, having an indexed orientation for each grain enables a second, more informative step — grain refinement — which yields intra-grain quantities, in particular the deviatoric strain tensor.

The segmentation produces 2067 grain candidates. For practicality, refinement is restricted to the 200 candidates with the highest average peak intensity. Each corresponding Laue pattern is indexed with LaueTools to extract an orientation matrix. Automatic indexation succeeds for 173 patterns, while 27 require manual intervention using the LaueTools graphical user interface; among these, 17 additional orientations are recovered, leaving 10 patterns that cannot be indexed. At this stage, equivalent orientations are filtered consistently with the cubic symmetry of the crystal structure.

The presence of duplicated orientations is expected and can be traced back to the segmentation choices. Using relatively strict similarity thresholds can split peaks originating from the same physical grain into multiple “grains” with slightly different appearance maps—for instance, separating subsets dominated by high-energy versus low-energy reflections. These split candidates carry different peak subsets, yet they often index to the same orientation matrix, producing duplicates. A second consequence is that some split subsets may contain too few (or too incomplete) reflections for robust indexation, which

plausibly contributes to the 10 non-indexed patterns (although a complete segmentation failure for some of them cannot be excluded).

After removing symmetry-equivalent and duplicated solutions, 86 distinct orientations remain. For each of them, the corresponding indexed pattern is searched back in the original set of 10,201 peak lists used for segmentation; when it is found with sufficient frequency and quality, strain refinement is performed. Two of the 86 orientations are discarded at this stage because they are supported only by a handful of matches in the full dataset and show a poor mean pixel deviation, indicating an unreliable solution. The final outcome is therefore 84 well-indexed grains for which intra-grain deviatoric strain could be refined. One representative deviatoric strain map is shown in Fig. 7 of the main text, and the set of refined orientations is used for the orientation statistics in Fig. 8.

Figure S7 summarizes the refined set by reporting, for each of the 84 grains, the number of indexed peaks across the scan. The distributions provide a compact representation of both the effective footprint of each grain (its appearance map) and the robustness of indexing: in most cases, the central regions consistently yield on the order of ~ 60 indexed reflections. Notably, several grains display very similar spatial footprints but distinct distributions of indexed peaks, a signature consistent with twinning. Inspection of their indexed patterns reveals a shared zone axis, confirming that these cases correspond to crystallographic twins rather than segmentation artefacts.

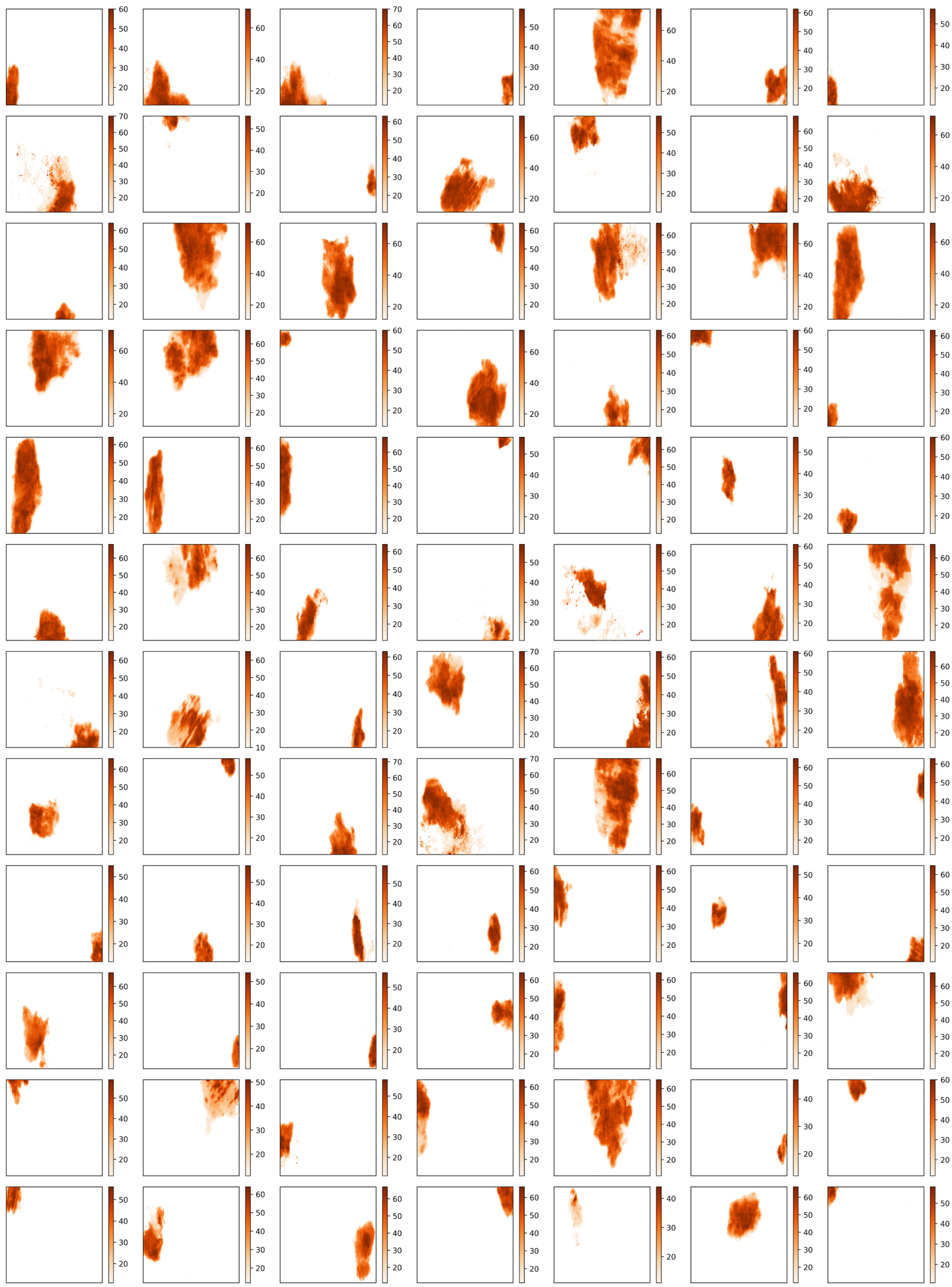

Figure S7. Maps of the number of indexed reflections across the scan for the 84 grains retained after validation and refinement (one panel per grain). Color encodes the number of peaks successfully indexed at each raster position, providing a representation of the grain footprint (appearance map) and for indexation robustness. Most grains show a central region with ~60 indexed reflections, while a subset exhibits similar spatial footprints but different indexed-peak distributions, consistent with twinning (shared zone axis in the corresponding indexed patterns).

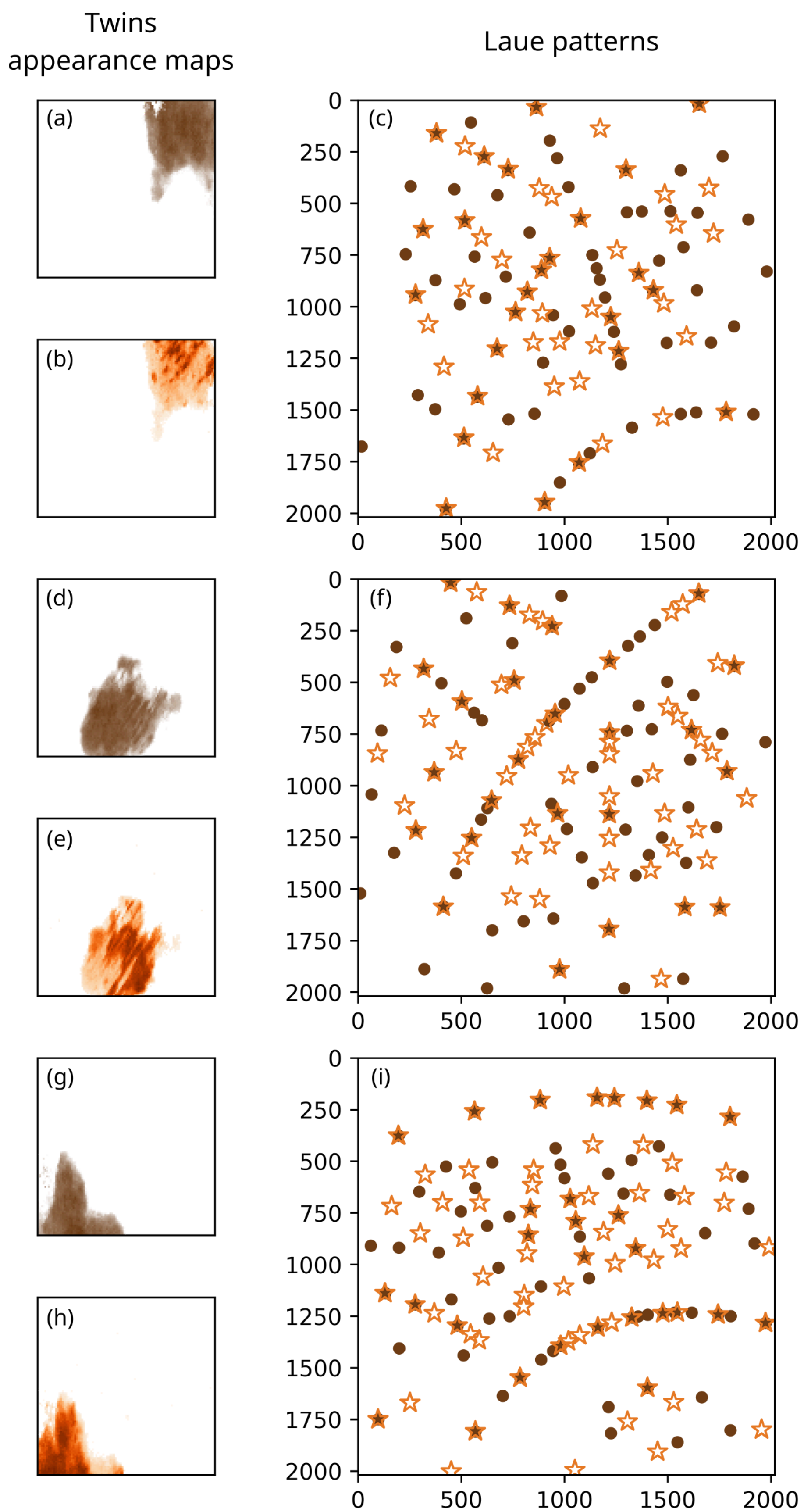

Figure S8. Three pairs of crystallographic twins. Each row contains the appearance maps, weighted by the number of indexed spots, and their respective superimposed Laue patterns. The appearance maps feature different densities of indexed spots and their pattern share a common zone axis.

S7. Validation on synthetic dataset with known ground truth

This section runs the full pipeline on a simulation where grain positions, sizes and orientations are known and compares the recovered microstructure against that ground truth.

```
from pathlib import Path
from itertools import repeat
from functools import partialmethod
import multiprocessing as mp
import numpy as np
np.random.seed(42)
np.set_printoptions(precision=3)
from time import time
from tqdm import tqdm
from graintools.simulation import Dataset
from graintools import segmentation as seg

from LaueTools.LaueGeometry import calc_uflab
from LaueTools.IOLaueTools import writefile_cor
from LaueTools.indexingSpotsSet import spotsset

from lauexplore import FitFile

nb_cpus = mp.cpu_count()

# ---------------------------------------------------------------------------- #
# Silence LaueTools' internal tqdm bars (LUT generation, etc.) inside workers.
# IndexSpotsSet creates progress bars that verbose=0 does not suppress. We
# disable tqdm globally *in the worker process* so those bars never render,
# while the parent-process bar we add in main() stays active.
# ---------------------------------------------------------------------------- #
def _silence_lauetools_tqdm():
    import tqdm as _tqdm_mod
    # Force disable=True on every tqdm instance created in this process.
    _tqdm_mod.tqdm.__init__ = partialmethod(
        _tqdm_mod.tqdm.__init__, disable=True
    )
    # LaueTools may import tqdm.auto; patch that binding too if present.
    try:
        import tqdm.auto as _tqdm_auto
        _tqdm_auto.tqdm.__init__ = partialmethod(
            _tqdm_auto.tqdm.__init__, disable=True
        )
    except Exception:
        pass


def _pool_init():
    """Worker initializer: run once per worker process."""
    _silence_lauetools_tqdm()
```

```python
MATERIAL = "3H-SiC"     # = 3C-SiC in the LaueTools dict (known mislabel)
EMIN, EMAX = 5, 25

calibration = {
    'dd': 80,
    'xcen': 1009,
    'ycen': 1008,
    'xbet': 0,
    'xgam': 0,
    'pixelsize': 0.0734,
    'xpixelsize': 0.0734,
    'ypixelsize': 0.0734,
    'CCDLabel': 'sCMOS',
    'framedim': np.array([2018., 2016.]),
    'detectordiameter': 148.12120000000002,
    'kf_direction': 'Z>0',
    'CCDCalibParameters': [80, 1009, 1008, 0, 0]
}

calibration_params = calibration["CCDCalibParameters"]

# --------------------------------------------------------------------------- #
# lauetools validation
# --------------------------------------------------------------------------- #

# Output directories
CWD = Path.cwd()
COR_DIR = CWD / "SEG_INDEX" / "CORFILES"
FIT_DIR = CWD / "SEG_INDEX" / "FITFILES"
for d in (COR_DIR, FIT_DIR):
    d.mkdir(parents=True, exist_ok=True)

# --------------------------------------------------------------------------- #
# 1. Write a proper .cor file from an (X, Y[, I]) peaklist
# --------------------------------------------------------------------------- #
def write_cor_from_peaklist(peaklist, calibration, cor_path):
    """Convert a segmentation peaklist to a LaueTools .cor file.

    The .cor file stores (2theta, chi, X, Y, I) AND the 5 calibration
    parameters in its header. IndexSpotsSet reads the geometry from that header,
    so it must be written via writefile_cor (do not hand-format it).

    Parameters
    ----------
    peaklist : (N, >=2) array. Columns 0,1 = pixel X, Y. Column 2 = intensity
               if present, otherwise a constant is used.
    calibration : the calib dict above.
    cor_path : Path or str, output path WITHOUT extension (LaueTools adds .cor).

    Returns the full path to the written .cor file.
    """
    peaklist = np.asarray(peaklist, dtype=float)
    x, y = peaklist[:, 0], peaklist[:, 1]

    # calc_uflab returns (2theta, chi). Some LaueTools versions accept
    # kf_direction as a kwarg, others infer it; fall back if it is rejected.
```

```python
    try:
        twotheta, chi = calc_uflab(
            x, y,
            calibration["CCDCalibParameters"],
            pixelsize=calibration["pixelsize"],
            kf_direction=calibration["kf_direction"],
        )
    except TypeError:
        twotheta, chi = calc_uflab(
            x, y,
            calibration["CCDCalibParameters"],
            pixelsize=calibration["pixelsize"],
        )

    if peaklist.shape[1] > 2:
        intensity = peaklist[:, 2]
    else:
        intensity = np.full_like(x, 1000.0)

    cor_path = Path(cor_path)
    prefix = cor_path.with_suffix("")          # writefile_cor appends ".cor"

    writefile_cor(
        str(prefix),
        twotheta, chi, x, y, intensity,
        param=calibration,   # the 5 calib params -> header
        sortedexit=False,
        overwrite=1,
        data_props=None,
        rectpix=0.0,
        dirname_output=None,
        verbose=0,
    )

    out = prefix.with_suffix(".cor")
    return out


# ---------------------------------------------------------------------------- #
# 2. Index the .cor file through the standard pipeline -> .fit file
# ---------------------------------------------------------------------------- #
def index_cor_file(cor_path, calibration=calibration, fit_dir=FIT_DIR,
                   previous_orientation=None, verbose=0):
    """Index one .cor file with IndexSpotsSet, writing a .fit file.

    This mirrors the standard single-crystal pipeline: the pattern is already
    isolated by segmentation, so we look for a single grain and refine through
    tightening angular tolerances.

    Returns the spotsset object (so you can inspect results in the notebook).
    """
    cor_path = Path(cor_path)

    prev = None
    if previous_orientation is not None:
        prev = [1, [np.asarray(previous_orientation)], 2, 2]
```

```python
    ss = spotsset()
    ss.importdatafromfile(str(cor_path))

    refinement = {
        'AngleTolLUT': 0.5,
        'nlutmax': 5,
        'list matching tol angles': [0.5, 0.2],
        'central spots indices': list(range(ss.nbspots-1)),
        'NBMAXPROBED': ss.nbspots,
        'MATCHINGRATE_ANGLE_TOL': 0.2,
        'MinimumMatchingRate': 5,
        'MinimumNumberMatches': 10,
        'UseIntensityWeights': False,
        'nbSpotsToIndex': 10000,
        'MATCHINGRATE_THRESHOLD_IAL': 2,
    }

    if hasattr(ss, "useinternalmultiprocessing"):
        ss.useinternalmultiprocessing = False

    ss.IndexSpotsSet(
        str(cor_path),
        MATERIAL,
        EMIN, EMAX,
        refinement,
        None,
        IMM=False,
        n_LUT=5,
        LUT=None,
        angletol_list=[0.5, 0.2],
        nbGrainstoFind=1,
        dirnameout_fitfile=str(fit_dir),
        corfilename=cor_path.name,
        verbose=verbose,
        MatchingRate_List=[2, 2, 2, 2, 2],
        previousResults=prev
    )
    return ss

# --------------------------------------------------------------------------- #
# 3. Convenience wrapper: peaklist -> .cor -> .fit in one call
# --------------------------------------------------------------------------- #
def index_peaklist(peaklist, calibration, name="grain_000",
                   previous_orientation=None, verbose=0):
    """Full standard path for one segmentation pattern.

    FIX 2 (robustness): never let one bad grain kill the whole pool. Catch and
    report, so the cluster job completes and the summary still runs.
    """
    try:
        cor_path = write_cor_from_peaklist(peaklist, calibration, COR_DIR / name)
        index_cor_file(
            cor_path, calibration=calibration, fit_dir=FIT_DIR,
            previous_orientation=previous_orientation, verbose=verbose,
        )
```

```python
    except Exception as e:
        # Keep going; the missing .fit is handled in the summary loop.
        print(f"[{name}] indexing failed: {type(e).__name__}: {e}")

def _index_peaklist_star(args):
    """Unpack a single tuple so the function works with Pool.imap_unordered."""
    return index_peaklist(*args)

def main():
    print("Generating dataset...", end="\r")
    t0 = time()
    data = Dataset((0, 0), (50, 50), (0.5, 0.5))
    data.generate_grains()
    data.simulate(MATERIAL, calibration_params, orientation_noise=0.005)
    dt = round(time() - t0, 1)

    total_peaks = sum(len(spot_list) for spot_list in data.spots)
    print(f"Generating dataset... Done ({dt} s)")
    # FIX 3: add \n separators so values do not run together.
    print(f"Number of grains: {len(data.grains)}")
    print(f"Number of simulated peaks: {total_peaks}")

    print("Starting segmentation...", end="")
    t0 = time()
    LUT = seg.buildLUT(data.spots, tol=1.25, workers=nb_cpus)
    spot_families = seg.track_positions(data.spots, LUT, tol=1.75, workers=nb_cpus)
    binary_images = np.array(
        [seg.amaps.to_binary_image(family[:, 0], data.shape) for family in
spot_families]
    )
    largest_ccs = np.array([seg.get_largest_cc(image) for image in binary_images])
    result_unfiltered = seg.find_matches(largest_ccs, 0.8)

    # NOTE: this counts peaks in clusters that get discarded for being too small.
    discarded_peaks = sum(len(r) for r in result_unfiltered if len(r) < 6)
    result_filtered = [res for res in result_unfiltered if len(res) >= 6]

    result = seg.result.build(spot_families, result_filtered, data.shape,
workers=nb_cpus)
    seg.result.write_toh5("simulation.h5", result)
    dt = round(time() - t0, 1)

    print(f"Done ({dt} s)")
    # FIX 3: separators here too.
    print(f"Number of segmented grains before filter: {len(result_unfiltered)}")
    print(f"Number of segmented grains after filter:  {len(result_filtered)}")
    print(
        f"Number of discarded peaks: {discarded_peaks} "
        f"({round(discarded_peaks / total_peaks * 100, 5)}%)"
    )


    peaklists = [r[1] for r in result]
    funcargs = list(zip(
        peaklists,
        repeat(calibration),
```

```python
        [f"grain_{i:0>3d}" for i in range(len(peaklists))],
    ))

    # initializer silences LaueTools' internal bars in each worker; the tqdm
    # below runs in the parent and ticks as each grain finishes. imap_unordered
    # yields results as they complete, which is what lets the bar advance.
    #with mp.Pool(nb_cpus, initializer=_pool_init) as pool:
    #    for _ in tqdm(
    #        pool.imap_unordered(_index_peaklist_star, funcargs, chunksize=1),
    #        total=len(funcargs),
    #        desc="Indexing grains",
    #    ):
    #        pass

    # ------------------------------------------------------------------ #
    # Summary
    # ------------------------------------------------------------------ #
    unindexed_percentages = []
    unindexed_peaks = []
    n_failed_fits = 0

    for i, peaklist in enumerate(peaklists):
        fit_path = FIT_DIR / f"grain_{i:0>3d}_g0.fit"

        # FIX 2: handle grains that produced no .fit (indexing failed).
        if not fit_path.exists():
            n_failed_fits += 1
            print(f"Grain {i: >3}: no .fit produced (indexing failed)")
            unindexed_percentages.append(100.0)
            unindexed_peaks.append(len(peaklist))
            continue

        ff = FitFile(fit_path)
        # FIX 1: consistent attribute name (was nb_indexed_sposts).
        nb_idx = ff.number_indexed_spots
        indexed = round(nb_idx / len(peaklist) * 100, 2)
        print(f"Grain {i: >3}: indexed {nb_idx}/{len(peaklist)} spots ({indexed}%)")
        unindexed_percentages.append(100 - indexed)
        unindexed_peaks.append(len(peaklist) - nb_idx)

    print("\n--- Summary ---")
    print(f"Grains processed:        {len(peaklists)}")
    print(f"Grains without a .fit:   {n_failed_fits}")
    print(f"Total unindexed peaks:   {np.sum(unindexed_peaks)}")
    print(f"Mean unindexed fraction: {np.mean(unindexed_percentages):.2f}%")

if __name__ == '__main__':
    main()
```

Outputs

```
Generating dataset... Done (54.2 s)
Number of grains: 169
Number of simulated peaks: 1203282
Starting segmentation... Done (43.1 s)
```

(the LUT has ~ 11000 peaks instead)

```
Number of segmented grains before filter: 195
Number of segmented grains after filter: 169
Number of discarded peaks: 34 (0.00283%)
Grain 0: indexed 75/76 spots (98.68%)
Grain 1: indexed 68/71 spots (95.77%)
Grain 2: indexed 72/73 spots (98.63%)
Grain 3: indexed 68/69 spots (98.55%)
Grain 4: indexed 68/68 spots (100.0%)
Grain 5: indexed 73/73 spots (100.0%)
Grain 6: indexed 68/69 spots (98.55%)
Grain 7: indexed 73/73 spots (100.0%)
Grain 8: indexed 69/70 spots (98.57%)
Grain 9: indexed 75/76 spots (98.68%)
Grain 10: indexed 70/71 spots (98.59%)
Grain 11: indexed 68/68 spots (100.0%)
Grain 12: indexed 71/71 spots (100.0%)
Grain 13: indexed 69/70 spots (98.57%)
Grain 14: indexed 71/72 spots (98.61%)
Grain 15: indexed 73/74 spots (98.65%)
Grain 16: indexed 73/74 spots (98.65%)
Grain 17: indexed 73/75 spots (97.33%)
Grain 18: indexed 66/67 spots (98.51%)
Grain 19: indexed 73/74 spots (98.65%)
Grain 20: indexed 65/65 spots (100.0%)
Grain 21: indexed 72/72 spots (100.0%)
Grain 22: indexed 71/72 spots (98.61%)
Grain 23: indexed 73/74 spots (98.65%)
Grain 24: indexed 72/75 spots (96.0%)
Grain 25: indexed 69/69 spots (100.0%)
Grain 26: indexed 69/70 spots (98.57%)
Grain 27: indexed 72/74 spots (97.3%)
Grain 28: indexed 77/77 spots (100.0%)
Grain 29: indexed 71/73 spots (97.26%)
Grain 30: indexed 69/70 spots (98.57%)
Grain 31: indexed 73/74 spots (98.65%)
Grain 32: indexed 71/71 spots (100.0%)
Grain 33: indexed 68/69 spots (98.55%)
Grain 34: indexed 72/73 spots (98.63%)
Grain 35: indexed 72/72 spots (100.0%)
Grain 36: indexed 68/69 spots (98.55%)
Grain 37: indexed 68/68 spots (100.0%)
Grain 38: indexed 75/74 spots (101.35%)
Grain 39: indexed 72/71 spots (101.41%)
Grain 40: indexed 64/75 spots (85.33%)
Grain 41: indexed 68/75 spots (90.67%)
Grain 42: indexed 74/64 spots (115.62%)
Grain 43: indexed 69/68 spots (101.47%)
Grain 44: indexed 73/73 spots (100.0%)
Grain 45: indexed 69/70 spots (98.57%)
Grain 46: indexed 67/75 spots (89.33%)
Grain 47: indexed 74/70 spots (105.71%)
```

```
Grain 48: indexed 71/73 spots (97.26%)
Grain 49: indexed 74/70 spots (105.71%)
Grain 50: indexed 66/69 spots (95.65%)
Grain 51: indexed 73/74 spots (98.65%)
Grain 52: indexed 71/73 spots (97.26%)
Grain 53: indexed 70/75 spots (93.33%)
Grain 54: indexed 66/74 spots (89.19%)
Grain 55: indexed 72/66 spots (109.09%)
Grain 56: indexed 70/73 spots (95.89%)
Grain 57: indexed 72/72 spots (100.0%)
Grain 58: indexed 68/72 spots (94.44%)
Grain 59: indexed 74/67 spots (110.45%)
Grain 60: indexed 71/73 spots (97.26%)
Grain 61: indexed 72/72 spots (100.0%)
Grain 62: indexed 74/75 spots (98.67%)
Grain 63: indexed 70/71 spots (98.59%)
Grain 64: indexed 72/73 spots (98.63%)
Grain 65: indexed 77/78 spots (98.72%)
Grain 66: indexed 74/75 spots (98.67%)
Grain 67: indexed 76/77 spots (98.7%)
Grain 68: indexed 68/69 spots (98.55%)
Grain 69: indexed 74/74 spots (100.0%)
Grain 70: indexed 67/68 spots (98.53%)
Grain 71: indexed 70/70 spots (100.0%)
Grain 72: indexed 71/71 spots (100.0%)
Grain 73: indexed 71/74 spots (95.95%)
Grain 74: indexed 73/73 spots (100.0%)
Grain 75: indexed 65/65 spots (100.0%)
Grain 76: indexed 71/71 spots (100.0%)
Grain 77: indexed 74/75 spots (98.67%)
Grain 78: indexed 72/73 spots (98.63%)
Grain 79: indexed 68/68 spots (100.0%)
Grain 80: indexed 78/78 spots (100.0%)
Grain 81: indexed 73/74 spots (98.65%)
Grain 82: indexed 77/77 spots (100.0%)
Grain 83: indexed 78/78 spots (100.0%)
Grain 84: indexed 69/70 spots (98.57%)
Grain 85: indexed 73/73 spots (100.0%)
Grain 86: indexed 69/69 spots (100.0%)
Grain 87: indexed 73/73 spots (100.0%)
Grain 88: indexed 78/78 spots (100.0%)
Grain 89: indexed 70/70 spots (100.0%)
Grain 90: indexed 69/69 spots (100.0%)
Grain 91: indexed 71/71 spots (100.0%)
Grain 92: indexed 73/75 spots (97.33%)
Grain 93: indexed 71/73 spots (97.26%)
Grain 94: indexed 78/78 spots (100.0%)
Grain 95: indexed 66/68 spots (97.06%)
Grain 96: indexed 72/74 spots (97.3%)
Grain 97: indexed 69/69 spots (100.0%)
Grain 98: indexed 74/75 spots (98.67%)
```

```
Grain 99: indexed 73/73 spots (100.0%)
Grain 100: indexed 74/75 spots (98.67%)
Grain 101: indexed 71/71 spots (100.0%)
Grain 102: indexed 70/70 spots (100.0%)
Grain 103: indexed 67/68 spots (98.53%)
Grain 104: indexed 73/76 spots (96.05%)
Grain 105: indexed 69/69 spots (100.0%)
Grain 106: indexed 75/75 spots (100.0%)
Grain 107: indexed 71/72 spots (98.61%)
Grain 108: indexed 77/78 spots (98.72%)
Grain 109: indexed 69/71 spots (97.18%)
Grain 110: indexed 72/73 spots (98.63%)
Grain 111: indexed 72/72 spots (100.0%)
Grain 112: indexed 70/71 spots (98.59%)
Grain 113: indexed 74/74 spots (100.0%)
Grain 114: indexed 78/78 spots (100.0%)
Grain 115: indexed 72/72 spots (100.0%)
Grain 116: indexed 70/70 spots (100.0%)
Grain 117: indexed 78/79 spots (98.73%)
Grain 118: indexed 69/69 spots (100.0%)
Grain 119: indexed 71/72 spots (98.61%)
Grain 120: indexed 70/72 spots (97.22%)
Grain 121: indexed 73/74 spots (98.65%)
Grain 122: indexed 76/76 spots (100.0%)
Grain 123: indexed 71/71 spots (100.0%)
Grain 124: indexed 78/78 spots (100.0%)
Grain 125: indexed 71/72 spots (98.61%)
Grain 126: indexed 68/69 spots (98.55%)
Grain 127: indexed 74/75 spots (98.67%)
Grain 128: indexed 74/74 spots (100.0%)
Grain 129: indexed 69/72 spots (95.83%)
Grain 130: indexed 79/80 spots (98.75%)
Grain 131: indexed 72/72 spots (100.0%)
Grain 132: indexed 68/70 spots (97.14%)
Grain 133: indexed 71/72 spots (98.61%)
Grain 134: indexed 74/75 spots (98.67%)
Grain 135: indexed 68/68 spots (100.0%)
Grain 136: indexed 69/70 spots (98.57%)
Grain 137: indexed 70/72 spots (97.22%)
Grain 138: indexed 69/69 spots (100.0%)
Grain 139: indexed 67/67 spots (100.0%)
Grain 140: indexed 72/74 spots (97.3%)
Grain 141: indexed 75/78 spots (96.15%)
Grain 142: indexed 72/73 spots (98.63%)
Grain 143: indexed 75/76 spots (98.68%)
Grain 144: indexed 72/72 spots (100.0%)
Grain 145: indexed 71/71 spots (100.0%)
Grain 146: indexed 69/70 spots (98.57%)
Grain 147: indexed 72/72 spots (100.0%)
Grain 148: indexed 75/75 spots (100.0%)
Grain 149: indexed 76/77 spots (98.7%)
```

```
Grain 150: indexed 68/70 spots (97.14%)
Grain 151: indexed 73/74 spots (98.65%)
Grain 152: indexed 75/77 spots (97.4%)
Grain 153: indexed 68/68 spots (100.0%)
Grain 154: indexed 74/74 spots (100.0%)
Grain 155: indexed 74/74 spots (100.0%)
Grain 156: indexed 70/72 spots (97.22%)
Grain 157: indexed 67/67 spots (100.0%)
Grain 158: indexed 67/68 spots (98.53%)
Grain 159: indexed 70/72 spots (97.22%)
Grain 160: indexed 68/69 spots (98.55%)
Grain 161: indexed 72/73 spots (98.63%)
Grain 162: indexed 67/68 spots (98.53%)
Grain 163: indexed 71/72 spots (98.61%)
Grain 164: indexed 69/69 spots (100.0%)
Grain 165: indexed 73/73 spots (100.0%)
Grain 166: indexed 74/75 spots (98.67%)
Grain 167: indexed 71/71 spots (100.0%)
Grain 168: indexed 67/68 spots (98.53%)

--- Summary ---
Grains processed: 169
Grains without a .fit: 0
Total unindexed peaks: 135
Mean unindexed fraction: 1.07%
```

Reconstructed Laue patterns

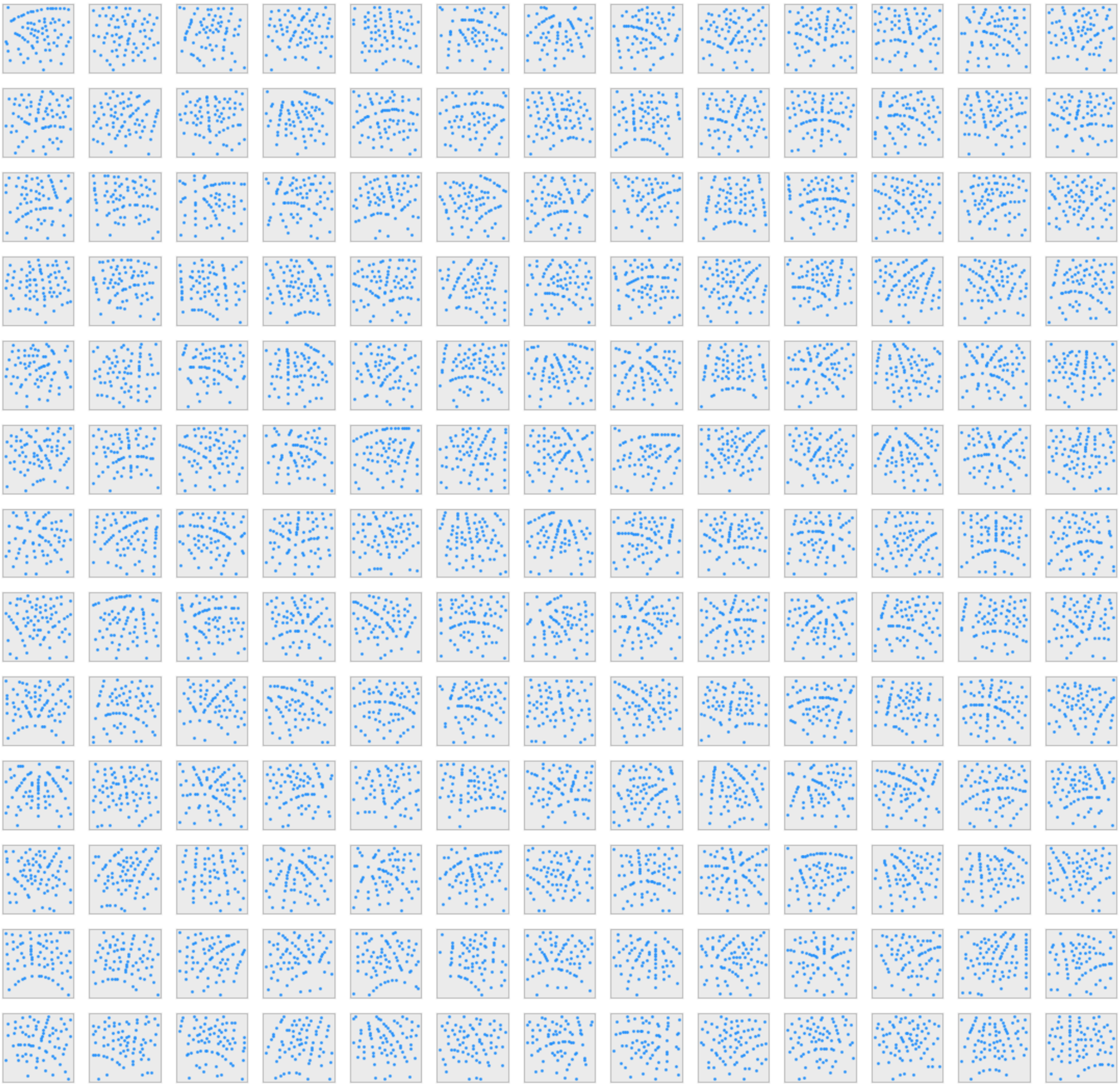

Reconstructed grain contours

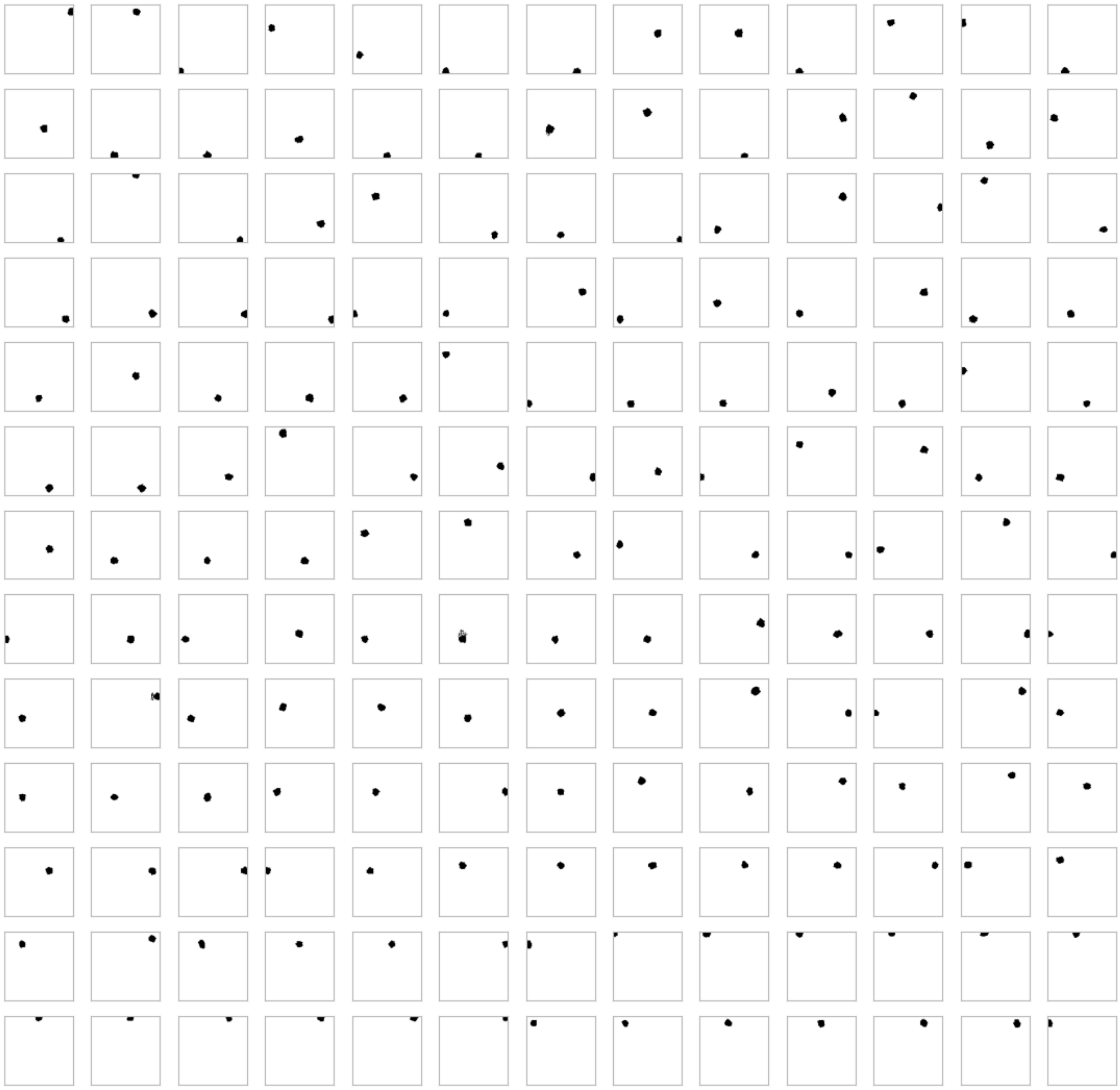

Supplement: Supplementary file 1 [file j-59-01584-sup1.pdf]
